# Serum deprivation initiates adaptation and survival to oxidative stress in prostate cancer cells

**DOI:** 10.1038/s41598-020-68668-x

**Published:** 2020-07-27

**Authors:** ElShaddai Z. White, Nakea M. Pennant, Jada R. Carter, Ohuod Hawsawi, Valerie Odero-Marah, Cimona V. Hinton

**Affiliations:** 1 Department of Biological Sciences, Clark Atlanta University (CAU), Atlanta, GA 30314 USA; 2 Center for Cancer Research and Therapeutic Development, Clark Atlanta University (CAU), Atlanta, GA 30314 USA; 3 Department of Molecular and Human Genetics, Baylor College of Medicine, Houston, TX 77030 USA

**Keywords:** Cancer microenvironment, Prostate

## Abstract

Inadequate nutrient intake leads to oxidative stress disrupting homeostasis, activating signaling, and altering metabolism. Oxidative stress serves as a hallmark in developing prostate lesions, and an aggressive cancer phenotype activating mechanisms allowing cancer cells to adapt and survive. It is unclear how adaptation and survival are facilitated; however, literature across several organisms demonstrates that a reversible cellular growth arrest and the transcription factor, nuclear factor-kappaB (NF-κB), contribute to cancer cell survival and therapeutic resistance under oxidative stress. We examined adaptability and survival to oxidative stress following nutrient deprivation in three prostate cancer models displaying varying degrees of tumorigenicity. We observed that reducing serum (starved) induced reactive oxygen species which provided an early oxidative stress environment and allowed cells to confer adaptability to increased oxidative stress (H_2_O_2_). Measurement of cell viability demonstrated a low death profile in stressed cells (starved + H_2_O_2_), while cell proliferation was stagnant. Quantitative measurement of apoptosis showed no significant cell death in stressed cells suggesting an adaptive mechanism to tolerate oxidative stress. Stressed cells also presented a quiescent phenotype, correlating with NF-κB nuclear translocation, suggesting a mechanism of tolerance. Our data suggests that nutrient deprivation primes prostate cancer cells for adaptability to oxidative stress and/or a general survival mechanism to anti-tumorigenic agents.

## Introduction

One of the hallmarks of cancer is the deregulation of cellular energetics which allows tumor cells to survive in environments that often results in death of normal cells^1^. Men are frequently bombarded by many endogenous agents, such as inflammation and oxidative phosphorylation within the mitochondria, and exogenous agents, such as ultraviolet rays, chemotherapy drugs, and cigarette smoke, which lead to oxidative stress and ultimately contributes to prostate cancer^2,3^. Oxidative stress damage is also well implicated across various disease spectrums, such as Alzheimer’s disease, Parkinson’s disease, arthritis, diabetes, atherosclerosis, and is recognized as one of the most influential precursors linked to prostate cancer development and progression^4^. In response to oxidative stress, cancer cells activate adaptive survival mechanisms which induce an abundance of cellular outcomes allowing cancer cells to survive hostile environments^5^.

As a consequence of deregulated cellular energetics, excessive production of reactive oxygen species (ROS), mitochondrial dysfunction, an impaired antioxidant system, or a combination of these factors lead to oxidative stress within tumor cells^6^. In addition, nutrient deficiencies within solid tumors stimulate oxidative stress and contribute to cancer lesions by directly diminishing ATP production and an overproduction of ROS^7^. ROS inducing agents play an intricate part in increasing oxidative stress leading to physiological and pathological processes, including DNA damage, cell adhesion, and cell survival, all of which contribute to carcinogenesis^8^, advanced malignancy and a poor prognosis of tumors^6^. In fact, Takeuchi *et al.* revealed that increasing oxidative DNA damage in patients with diseases is associated with increased cancer risk^9^. In addition, redox alterations in cancer cells are multifaceted due to the many factors involved in redox regulation and stress responses as well as the addition of ROS-generating agents, which do not always lead to cell death^6,10,11^, suggesting a form of acculturation to stress. Compared to non-cancerous cells, tumor cells function with higher levels of endogenous oxidative stress in vitro and in vivo, which indicates that oxidative stress adaptation is necessary for malignant transformation of cancer cells, metastasis, and resistance to anticancer drugs^12,13^. Evidence also suggests that higher levels of ROS contribute to tumor progression and other diseases related to oxidative damage making ROS indispensable for cell survival and differentiation^14,15^.

Nutrient deprivation is a universal phenomenon in solid tumors due to poor and/or a competing blood supply, especially in the center of a tumor mass, during metastasis when cells disengage from the vasculature to move, and/or during therapy that is designed to collapse a vasculature or induce cell death^16,17^. When a tumor's growth exceeds its vascular supply, tumor cells must adapt to a lower availability of nutrients and oxygen resulting in a reversible cell growth arrest (quiescence)^18^. This quiescent phenotype is fundamental to tissue renewal and regeneration, as well as protecting against stress and toxicities, which is essential for long-lived cell types such as tumor and stem cells^19,20^. Quiescent cells typically express lower levels of Rb-E2F pathway activators (e.g., CycD, Cdk2) and higher levels of, p27^Kip1^, a Cdk inhibitor, and Mirk/DYRK1B, a cell cycle serine/threonine kinase which both play a role in increased tumor aggressiveness and poor patient outcome^21–25^. Tumor cells often experience quiescent periods during tumor development in which they are not proliferative but remain alive. In this state, they are unresponsive to chemotherapies and responsible for many cases of relapse^26^. Hence, there is difficulty in isolating these unique cells from patients due to limited understanding of cellular quiescence in cancer and the challenges in research development of therapies to prevent cancer relapse.

Adaptation is a challenge in which tumor cells must undergo to survive hostile environments, and consequently, becomes a major barrier for drug resistance. As such, transcription factors such as nuclear factor kappa-light-chain-enhancer of activated B cells (NF-κB) contribute to stress adaptation which occurs in response to oxidative stress and other types of stress leading to transformation, survival, and angiogenesis^8,27,28^. NF-κB is a dimer composed of the RelA (p65) and NF-κB1 (p50) or NF-κB2 (p52) subunits. In normal resting cells, NF-κB is sequestered in the cytoplasm through binding to IκB, and activation results in degradation of IκB and subsequent NF-κB release and translocation to the nucleus for binding to a target gene promoter^29,30^. NF-κB is often present during tumor initiation, apoptosis evasion, tumor angiogenesis, and metastasis, all of which are events that exhibit a level of cellular stress^31^.

Nutrient deficiencies are inevitable in solid tumors, but the full effect of cancer cell adaption to oxidative stress is not yet clear. Therefore, we sought to analyze how serum deprivation protects, or primes, tumor cells to manage oxidative stress. We observed that serum deprivation prevented an apoptotic phenotype in prostate cancer cells, and presented markers of quiescence, presumably, to manage oxidative stress. We also observed nuclear translocalization of RelA/p65 (NF-κB) during oxidative stress adaptation, and that this transcription factor was also essential for maintaining adaptation. Our data suggest that serum deprivation primes prostate tumor cells for oxidative stress facilitating survival through stressful conditions.

## Results

### Serum deprivation prevented an apoptotic phenotype in prostate cancer cells

**Figure 1:**
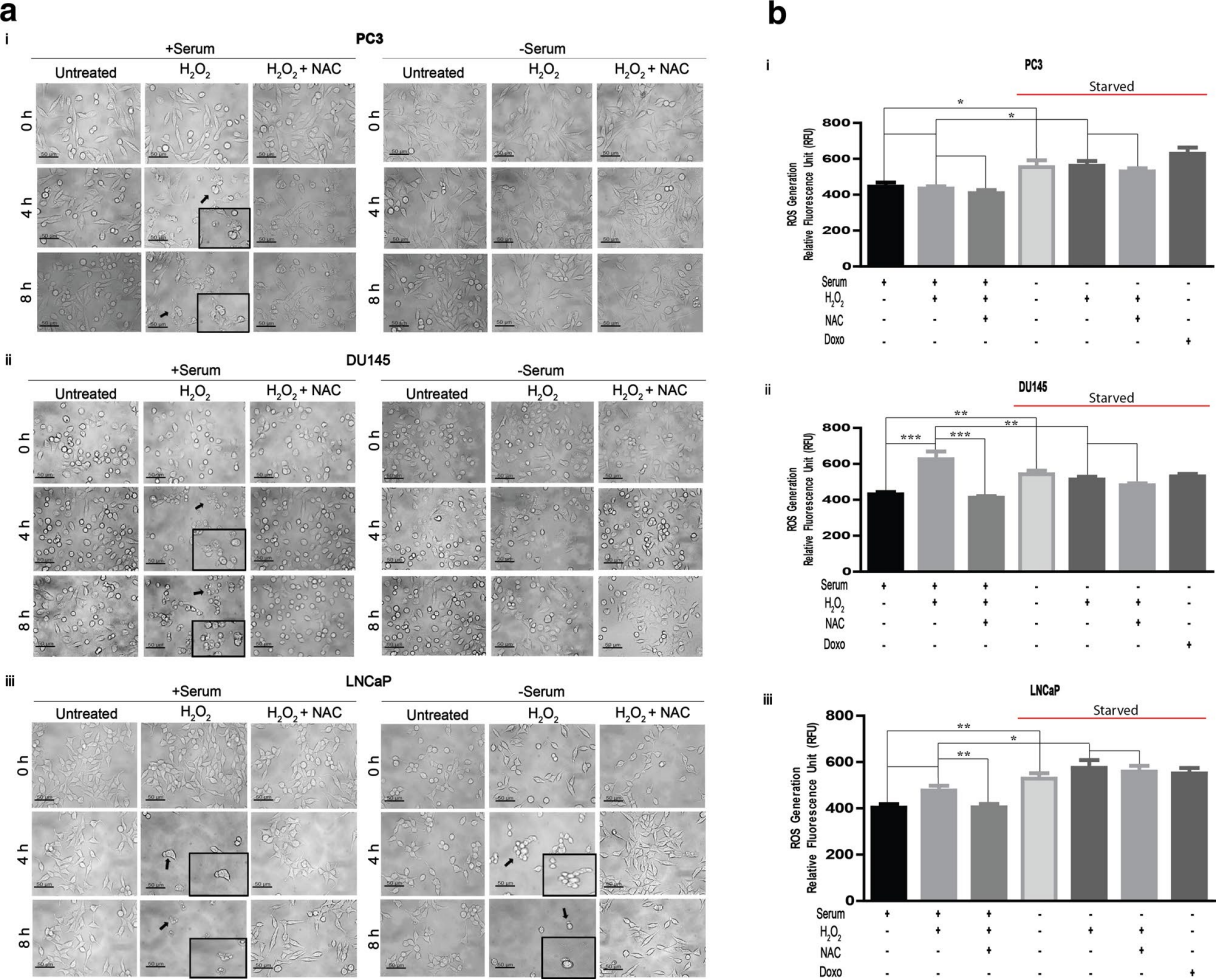

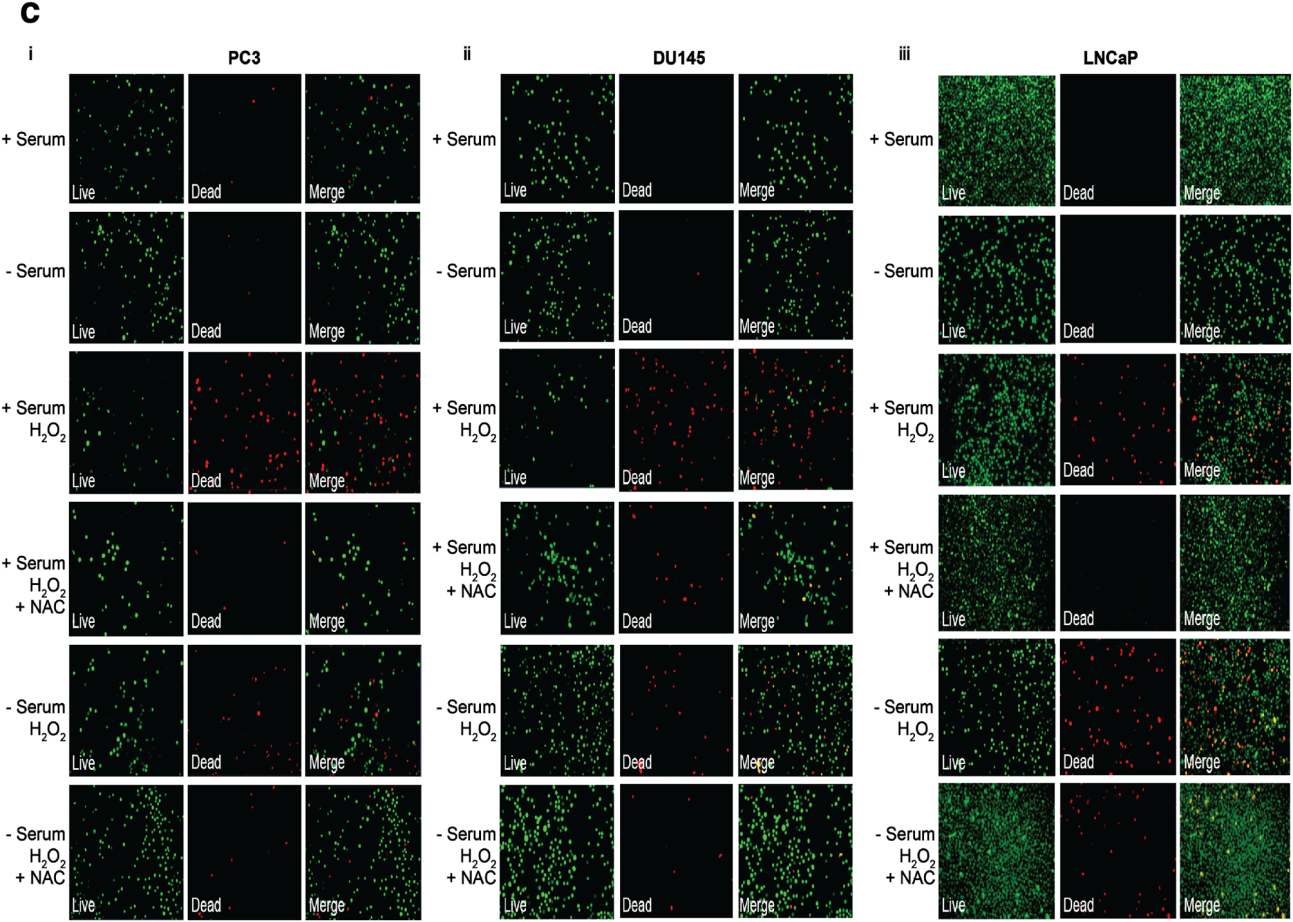
(**a**) **Serum deprivation prevents an apoptotic phenotype in prostate cancer cells.** One hundred fifty thousand (1.5 × 10^5^) PC3 (i), DU145 (ii), and LNCaP (iii) prostate cancer cell lines were plated in 6-well plates prior to serum deprivation and treatment with 5 mM n-acetyl-cysteine (NAC) for 1 h, and/or 250 μM H_2_O_2_ at various time points (0, 4, 8 h). Phase contrast microscopy (20×) was used to capture images. Arrows highlight areas of cells with a cell death phenotype. Scale bar = 50 μm. (**b**) **Serum deprivation generates ROS.** Ten thousand (1 × 10^4^) serum-containing and serum-deprived PC3 (i), DU145 (ii), and LNCaP (iii) prostate cancer cells were plated in black 96-well microplates. Cells were incubated for 1 h with 10 μM of 2′,7′-Dichlorofluorescin diacetate (DCFDA) followed by stimulation with 250 μM H_2_O_2_ alone, pre-treatment with NAC (5 mM) followed by stimulation with 250 μM H_2_O_2_, or 2 μM Doxorubicin (Doxo; positive control to induce ROS) for 2 h via a microplate reader to detect ROS generation. The mean ± SEM of data were obtained from four independent replicate experiments. Statistical analysis (*one-way ANOVA*) was performed with GraphPad Prism (***p < 0.001; **p < 0.01; *p < 0.05). (**c**) **Serum deprivation preserved viability during oxidative stress during.** Cell viability was measured in PC3 (i), DU145 (ii), and LNCaP (iii) prostate cancer cells. Two hundred thousand (2 × 10^5^) serum-containing and serum-deprived cells were either pre-treated with 5 mM n-acetyl-cysteine (NAC) and/or treated with 250 μM H_2_O_2_ for 4 h. Cell viability was measured via a live/dead cell assay (ThermoFisher) according to manufacturer’s protocol. Green = *live*; Red = *dead*).

Serum deprivation in vitro reduces levels of growth factors in tumor cells that could que cells for death^32^. However, Martindale *et al.* demonstrated that serum deprivation primes cells to adapt to injury, stress, or death^33^. To study the role of serum deprivation in adaptive survival during oxidative stress in prostate cancer, we first visualized the phenotype of serum-containing and serum-deprived prostate cancer cell lines, and the phenotype of serum-deprived cell lines stimulated with hydrogen peroxide (H_2_O_2_), our model of oxidative stress^34–36^. Serum-containing cells in each prostate cancer cell line (PC3, DU145 and LNCaP) were vulnerable to H_2_O_2_ exposure and displayed significant cell death morphology as early as 4 h as denoted by black arrows (Fig. 1a). Additionally, we observed morphological hallmarks which accompany apoptosis such as rounding up of the cell, pyknosis (reduction in cellular volume), nuclear shrinkage, and retraction of pseudopodia^37^. However, over time (up to 8 h), serum-deprived PC3 and DU145 cells remained viable and developed a more flat morphology in addition to an increase in cell size and number—a phenotype that was more apparent with the addition of H_2_O_2_^38^. In contrast, serum-deprived LNCaP cells (Fig. 1aiii) maintained a cell death morphology, and more so after H_2_O_2_ stimulation, which was inhibited by the antioxidant, N-acetyl cysteine (NAC). Particularly for the PC3 and DU145 cell lines, this data suggests that serum deprivation may induce an adaptation to oxidative stress and promote long-term survival in more aggressive cancer cells.

**Figure 2:**
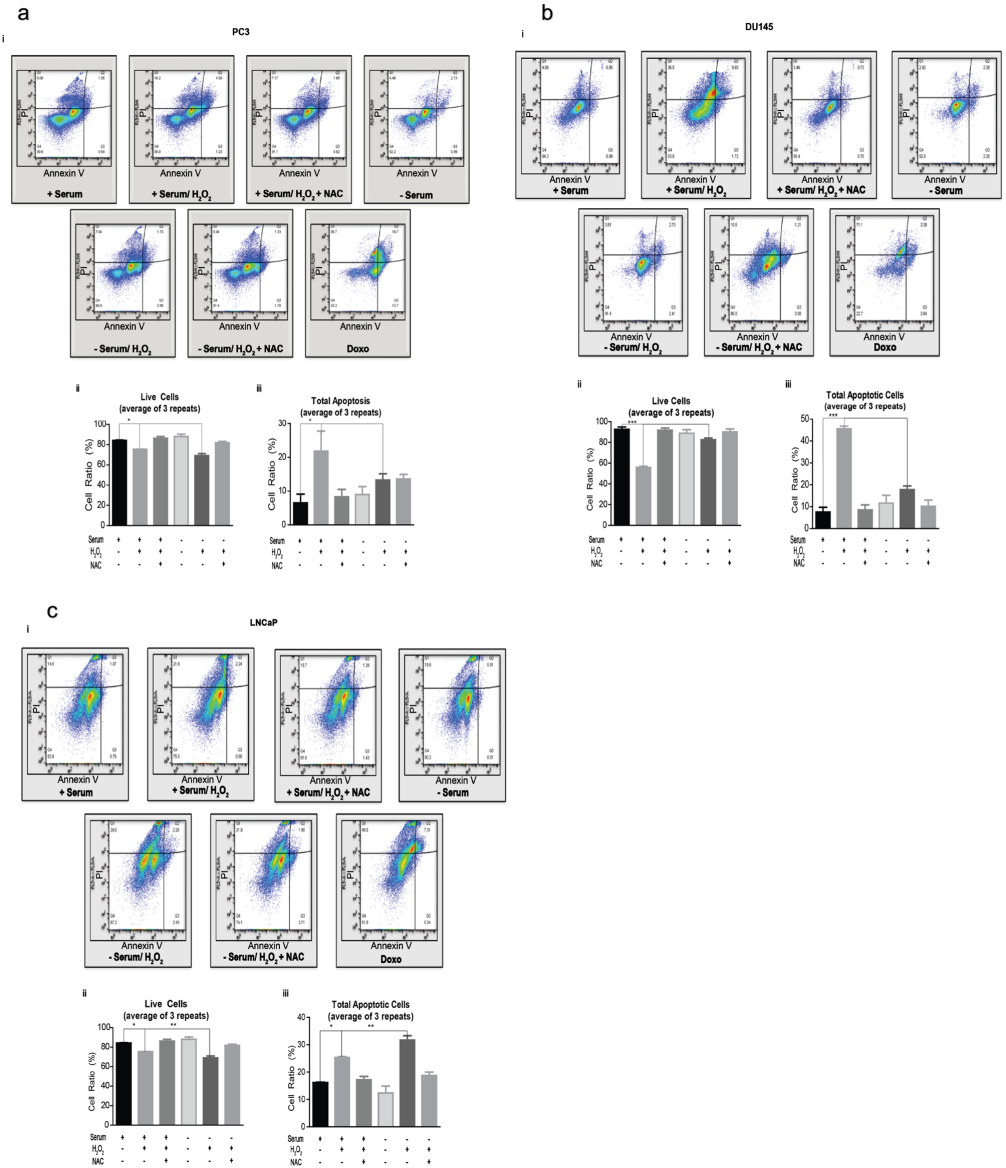
(**a**) **Serum deprivation protected cancer cells from apoptosis during oxidative stress.** (i) One million (1 × 10^6^) PC3 prostate cancer cells were harvested with or without serum, then were treated with 250 μM H_2_O_2_, and/or 10 mM n-acetyl-cysteine (NAC) or 2 μM Doxorubicin (Doxo). Apoptosis was measured via annexin V-FITC/PI double staining and flow cytometry. **Q1** (Annexin V+ , PI+): dead cells; **Q2** (Annexin V-, PI+): late stage apoptosis cells; **Q3** (Annexin V+, PI−): early stage apoptosis cells; and **Q4** (Annexin V− , PI−): live cells. (ii) A graphical representation of live cells. (iii) A graphical representation of total apoptotic cells (a sum of dead, early- and late-stages). Images were acquired via flow cytometry (Accuri C6 Cytometer; BD Biosciences); data was analyzed using FlowJo (v10). The mean ± SEM of data were obtained from three independent replicate experiments. Statistical was analysis (one-way ANOVA) was done with GraphPad Prism (***p < 0.001; **p < 0.01; *p < 0.05). (**b**) **Serum deprivation protected cancer cells from apoptosis during oxidative stress.** (i) One million (1 × 10^6^) DU145 prostate cancer cells were harvested with or without serum, then were treated with 250 μM H_2_O_2_, and/or 10 mM n-acetyl-cysteine (NAC) or 2 μM Doxorubicin (Doxo). Apoptosis was measured via annexin V-FITC/PI double staining and flow cytometry. **Q1** (Annexin V+ , PI+): dead cells; **Q2** (Annexin V−, PI+): late stage apoptosis cells; **Q3** (Annexin V+ , PI−): early stage apoptosis cells; and **Q4** (Annexin V− , PI−): live cells. (ii) A graphical representation of live cells. (iii) A graphical representation of total apoptotic cells (a sum of dead, early- and late-stages). Images were acquired via flow cytometry (Accuri C6 Cytometer; BD Biosciences); data was analyzed using FlowJo (v10). The mean ± SEM of data were obtained from three independent replicate experiments. Statistical analysis (*one-way ANOVA*) was done with GraphPad Prism (***p < 0.001; **p < 0.01; *p < 0.05). (**c**) **Serum deprivation protected cancer cells from apoptosis during oxidative stress.** (i) One million (1 × 10^6^) LNCaP prostate cancer cells were harvested with or without serum, then were treated with 250 μM H_2_O_2_, and/or 10 mM n-acetyl-cysteine (NAC) or 2 μM Doxorubicin (Doxo). Apoptosis was measured via annexin V-FITC/PI double staining and flow cytometry. **Q1** (Annexin V+ , PI+): dead cells; **Q2** (Annexin V−, PI+): late stage apoptosis cells; **Q3** (Annexin V+ , PI −): early stage apoptosis cells; and **Q4** (Annexin V− , PI−): live cells. (ii) A graphical representation of live cells. (iii) A graphical representation of total apoptotic cells (a sum of dead, early- and late-stages). Images were acquired via flow cytometry (Accuri C6 Cytometer; BD Biosciences); data was analyzed using FlowJo (v10). The mean ± SEM of data were obtained from three independent replicate experiments. Statistical analysis (*one-way ANOVA*) was done with GraphPad Prism (***p < 0.001; **p < 0.01; *p < 0.05).

Tumor cells can maintain higher ROS levels than normal cells, and are reported to confer more resistance to ROS-mediated death^39,40^. Serum deprivation, alone, induces oxidative stress^41^ which we measured in each cell line with 2′,7′-dichlorofluorescin diacetate (DCFDA) for ROS generation. Serum-deprived PC3, DU145, and LNCaP cell lines displayed a significant increase in the generation of ROS compared to serum-containing control cells (overall *p* values: PC3 (≤ 0.001); DU145 (≤ 0.001); LNCaP (≤ 0.067)) (Fig. 1b). The addition of H_2_O_2_ in serum-containing cells and serum-deprived cells displayed no significant change in ROS generation compared to their respective controls in PC3. However, this increase in ROS generation with the addition of H_2_O_2_ was observed only in serum-containing cells in DU145, but in both serum-containing and serum-deprived cells of LNCaP. As expected, pre-treatment with ROS-scavenger, NAC, prior to stimulation with H_2_O_2_, inhibited significant ROS generation. We note the observation of depriving cells of serum only increases ROS generation, and additional ROS stimulation did not increase ROS production. It is believed that serum withdrawal causes cells to stop growing and initiates apoptosis; however, we observed a morphology that is inconsistent with apoptosis in serum-deprived samples exposed to ROS^42,43^ (Fig. 1a). To determine whether serum deprivation primes cells for adaptive survival to increasing oxidative stress, we investigated the viability of cells grown under serum-deprived conditions. Our results indicate that PC3 and DU145 prostate cancer cells were viable when serum-deprived and maintained viability after exposure to H_2_O_2_ compared to serum-containing cells exposed to H_2_O_2_ (Fig. 1ci–ii). Conversely, LNCaP prostate cancer cells stimulated with H_2_O_2_ in both serum-containing and serum-deprived conditions were vulnerable to oxidative stress and died within 4 h of H_2_O_2_ stimulation (Fig. 1ciii). To quantify death, we examined apoptosis. We observed a significant increase in total apoptosis (a sum of dead, early- and late-stages) in serum-containing cells stimulated with H_2_O_2_ in each cell line (Fig. 2a–c). However, our assumptions that serum-deprivation primes cells for stress survival were supported by the observation that serum-deprived PC3 (Fig. 2a) and DU145 (Fig. 2b) cells displayed no significant apoptotic profile after exposure to H_2_O_2_. Consistent to what we observed in Fig. 1ciii, serum-containing and serum-deprived LNCaP cells exposed to H_2_O_2_ were vulnerable to death (Fig. 2c). In literature, LNCaP cells demonstrate low survivability upon serum withdrawal^44^. In our observation, LNCaP cells did not adapt to the onset of oxidative stress and have not served as a reliable model to investigate adaptability to oxidative stress. Thus, LNCaP prostate cancer cell line was not used in the forthcoming experiments.

### Quiescence is a consequence of serum-deprivation and helps to manage oxidative stress

**Figure 3:**
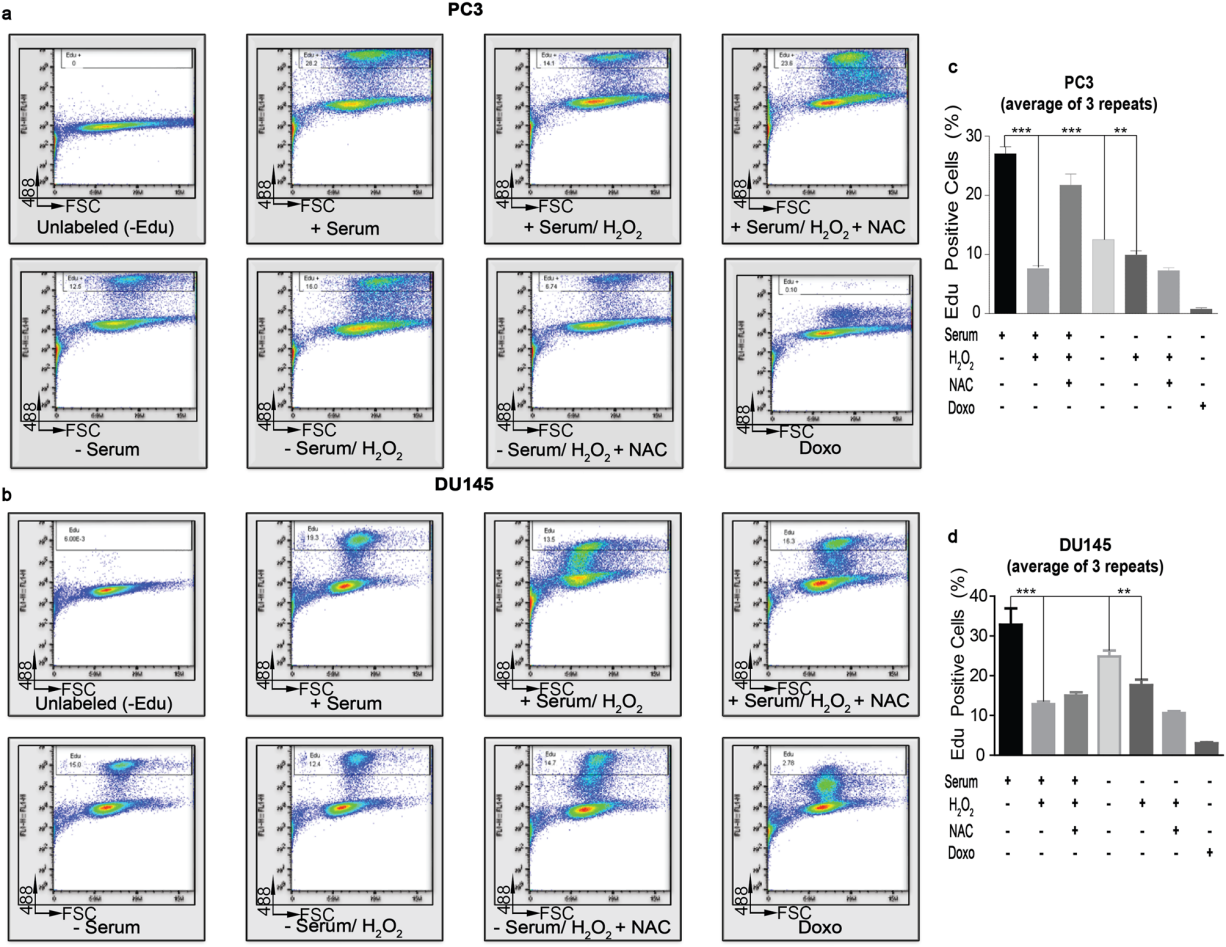
**Proliferation profile of serum-deprived cells.** Dot plot of EdU-488 staining (Y-axis, 488 vs FSC). One million (1.0 × 10^6^) serum-containing and serum-deprived PC3 (**a**) and DU145 (**b**) prostate cancer cells were treated with 250 μM H_2_O_2_, or co-treated with 10 mM n-acetyl-cysteine (NAC) or 2 μM Doxorubicin (Doxo) for 24 h. Cells were incubated with 20 μM EdU label per manufacturer’s instructions; control cells were cultured in 10% FBS without EdU. Images were acquired via flow cytometry (Accuri C6, BD Biosciences); data was analyzed using FlowJo (v10). The percentage of gated cells (EdU^+^) is highlighted. (**c**) A graphical representation of EdU^+^ PC3 cells. (d) A graphical representation of EdU^+^ DU145 cells. The mean ± SEM of data were obtained from three independent replicate experiments. Statistical analysis (*one-way ANOVA*) was done with GraphPad Prism (***p < 0.001; **p < 0.01; *p < 0.05).

Serum deprivation in vitro induces proliferation arrest to protect cells from toxicities; consequently, a reversible cell cycle arrest (quiescence) is associated with this phenotype and cellular consequences are multi-drug chemo-resistance and a propensity to evade apoptosis^18,45^. Therefore, we examined proliferation during oxidative stress via the 5-ethynyl-2′-deoxyuridine (EdU) assay. Serum-deprived PC3 (Fig. 3a) and DU145 (Fig. 3b) cells exhibited proliferation arrest, as expected, and continued after exposure to H_2_O_2_ for 24 h. Moreover, cell cycle arrest was also observed when cells were grown with serum and stimulated with H_2_O_2_. These observations simply report that a stress event (via loss of serum or OS) halts proliferation. Doxorubicin, a potent inducer of apoptosis, served as a control.

**Figure 4:**
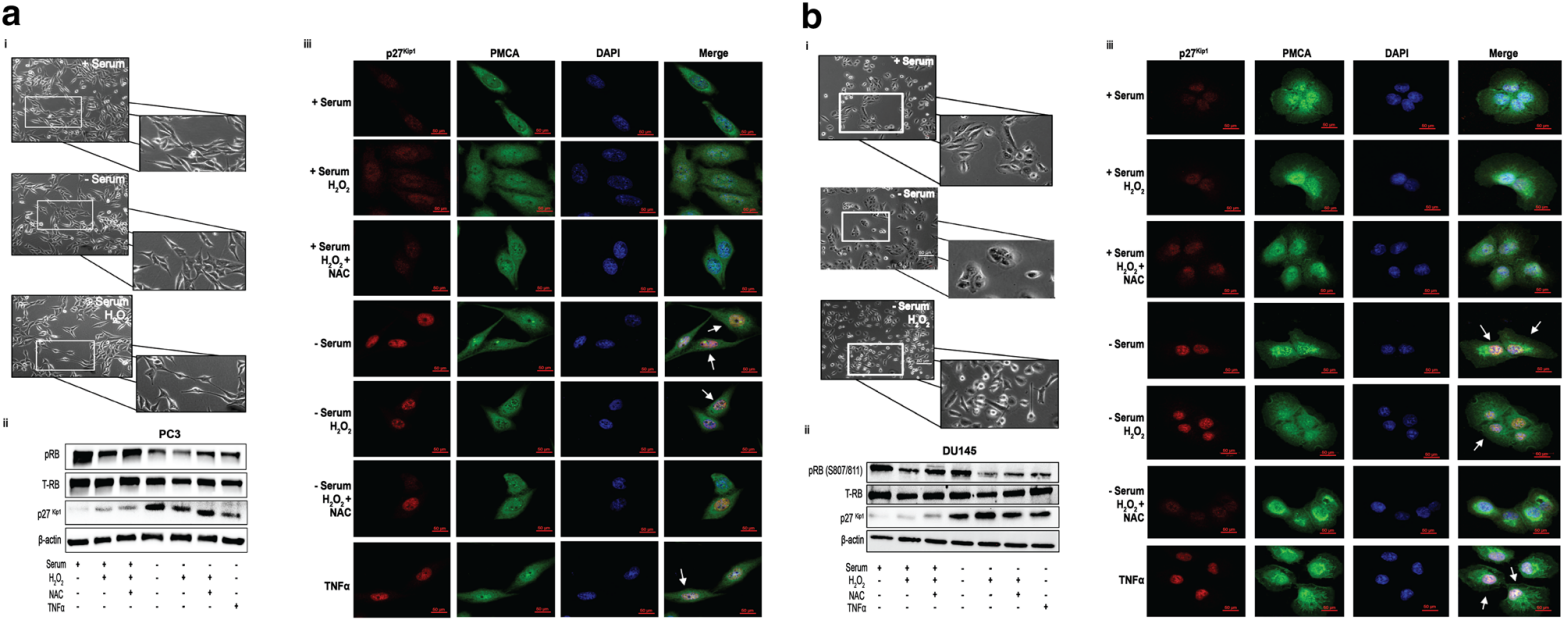
(**a**) **Serum-deprived cells present a quiescent phenotype to manage oxidative stress.** (i) One hundred fifty thousand (1.5 × 10^5^) PC3 prostate cancer cells were plated with or without serum (24 h) prior to treatment with 250 μM H_2_0_2_ for 4 h. Phase contrast microscopy (20×) was captured cell morphology. (ii) One million (1 × 10^6^) serum-containing and serum-deprived PC3 prostate cancer cells were pre-treated with 5 mM n-acetyl-cysteine (NAC) for 1 h prior to treatment with 250 μM H_2_O_2_. Quiescence was determined via Western blot analysis for phosphorylated-retinoblastoma (pRB) or p27^Kip1^ protein expression. β-actin served as a loading control. Pictures of gels/blots were cropped to focus on target protein expression. Full length gels/blots are included in supplementary figure 1. (iii) One hundred fifty thousand (1.5 × 10^5^) cells were treated as mentioned above and fixed in 4% paraformaldehyde prior to incubation with rabbit anti-p27^Kip1^ and mouse anti-PMCA antibodies, followed by Cy3-conjugated anti-rabbit and Alexa Fluor-488 anti-mouse antibodies. Imaging of cells (63×) was performed on a Zeiss LSM700 Confocal Microscope. Scale bar = 50 μm. (**b**) **Serum-deprived cells present a quiescent phenotype to manage oxidative stress.** (i) One million (1.0 × 10^6^) DU145 prostate cancer cells were plated with or without serum (24 h) prior to treatment with 250 μM H_2_0_2_ for 4 h. Phase contrast microscopy (20×) was utilized to captured cell morphology. (ii) One million (1 × 10^6^) serum-containing and serum-deprived DU145 prostate cancer cells were pre-treated with 5 mM n-acetyl-cysteine (NAC) for 1 h prior to treatment with 250 μM H_2_O_2_. Quiescence was determined via Western blot analysis for phosphorylated-retinoblastoma (pRB) or p27^Kip1^ protein expression. β-actin served as a loading control. Pictures of gels/blots were cropped to focus on target protein expression. Full length gels/blots are included in supplementary figure 2. (iii) One hundred fifty thousand (1.5 × 10^5^) DU145 prostate cancer cells were treated as mentioned above and fixed in 4% paraformaldehyde prior to incubation with rabbit anti-p27^Kip1^ and mouse anti-PMCA antibodies, followed by Cy3-conjugated anti-rabbit and Alexa Fluor-488 anti-mouse antibodies. Imaging of cells (63×) was performed on a Zeiss LSM-700 Confocal Microscope. Scale bar = 50 μm.

Since we did not observe apoptosis or proliferation in our serum-deprived cells, nor when further exposed to oxidative stress via H_2_O_2_, we explored whether epithelial tumor cells may temporarily transition to a quiescent phenotype for management and survival during serum deprivation and/or oxidative stress^46^. We examined the morphology of serum-deprived PC3 and DU145 cells alone or serum-deprived and stimulated with H_2_O_2_ for 1 h (Fig. 4ai, bi). It is reported, cells which display a shrunken, round, and flat morphology is indicative of a quiescent phenotype^18,33^. Indeed, we observed PC3 and DU145 cells cultured without serum, as well serum-deprived cells with H_2_O_2_, displayed a rounder and flatter phenotype compared to cells in serum suggesting that cells submit to a quiescent phenotype for survival and adaption to stress that may be a consequence of nutrient deprivation or downstream stress.

To confirm the quiescent phenotype on a molecular level, serum-deprived PC3 and DU145 prostate cancer cells were examined for expression of quiescent markers: (1) tumor suppressor gene, retinoblastoma (RB)^23,47^; and (2) CDK inhibitor, cyclin-dependent kinase inhibitor 1B (p27^Kip1^)^24^. It is well known that RB is an enforcer of quiescence^47^, and as such, the diminishing expression of phosphorylated-RB (pRB) in serum-deprived cells and serum-deprived cells exposed to H_2_O_2_ indicates the onset of quiescence under stress (Fig. 4aii,bii). Accordingly, an accumulation of p27^Kip1^ in the nucleus is indicative of G0/G1 arrest and quiescence ^48^, and an observation of strong p27^Kip1^ protein expression in the same samples further confirms that quiescence is necessary for cells to survive oxidative stress (Fig. 4aii,bii). Furthermore, we detected more accumulation of p27^Kip1^ in the nucleus of PC3 (Fig. 4aiii) and DU145 (Fig. 4biii) cells in samples that were starved and further exposed to H_2_O_2_ supporting that quiescence provides adaptive protection for tumor cells under stress to ensure long-term survival.

### RelA/p65 (NF-κB) translocates to the nucleus in response to oxidative stress adaptation

**Figure 5:**
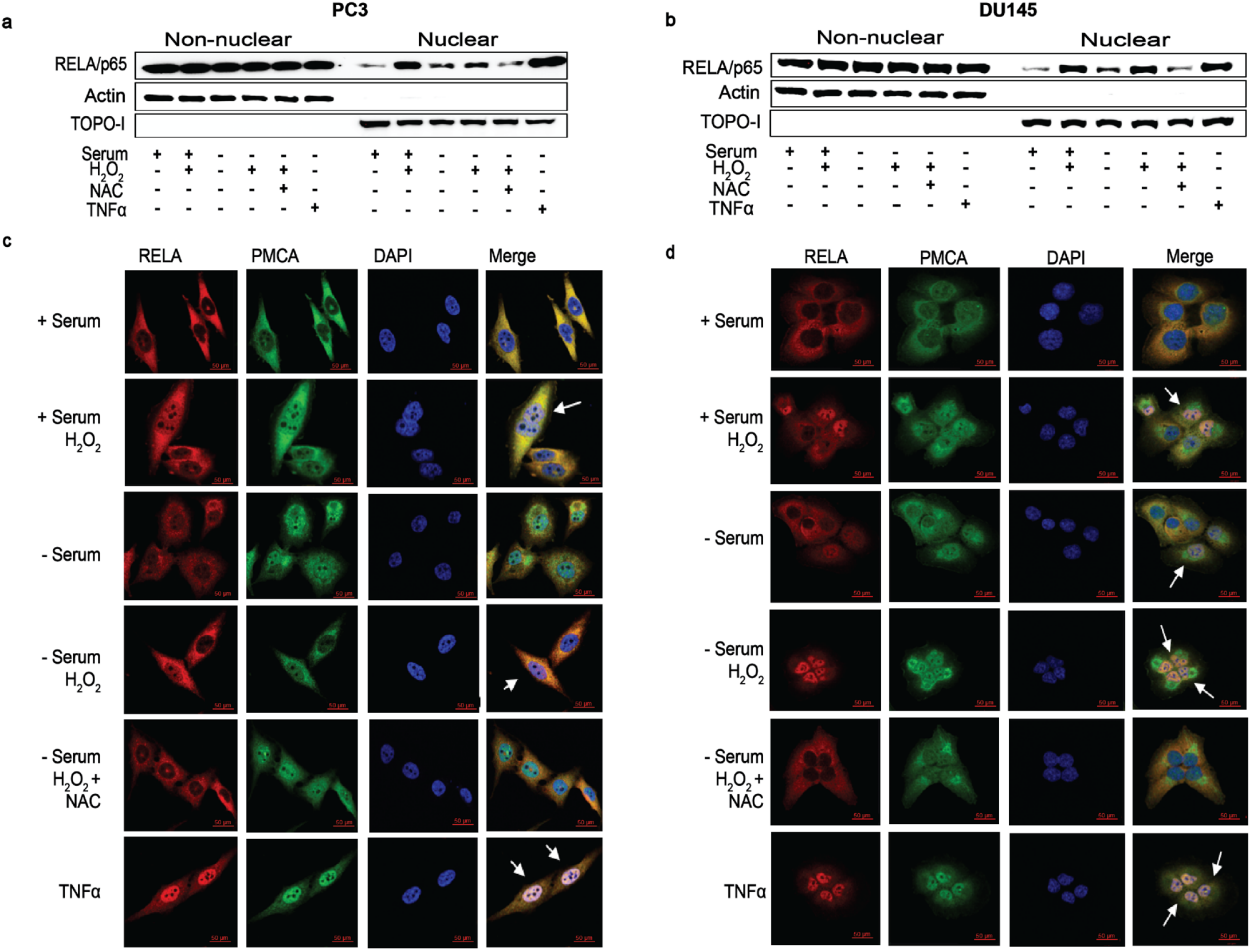
**RelA/p65 (NF-κB) translocates to the nucleus in response to oxidative stress.** (**a**, **b**) Two million (2 × 10^6^) PC3 and DU145 cells were harvested with or without serum for 24 h prior to pre-treatment with 5 mM n-acetyl-cysteine (NAC) for 1 h followed by 250 μM H_2_O_2_ for 1 h, or TNFα (0.1 ng/mL; 30 min). Proteins were harvested via subcellular fractionation according to the manufacturer’s instruction and resolved by Western blot analysis. Immunoblots were probed with anti-RelA/p65; anti-β-actin (non-nuclear) and anti-Topoisomerase1 (Topo 1, nuclear) served as markers for fractionation purity and as loading controls. Pictures of gel/blots were cropped to focus on target protein expression. Full length gels/blots are included in supplementary figure 3 (PC3) and 4 (DU145). (**c**, **d**) One hundred thousand (1 × 10^5^) PC3 and DU145 cells treated as mentioned above and were harvested for immunocytochemistry, fixed with 4% paraformaldehyde, blocked, then incubated with rabbit anti-RelA/p65 and mouse anti-PMCA antibodies, followed by Cy3-conjugated anti-rabbit and FITC-conjugated anti-mouse antibodies. Imaging of cells (63×) was performed on a Zeiss LSM-700 Confocal Microscope. Scale bar = 50 μm.

NF-κB is recognized as a redox-sensitive transcription factor and is a major player in the cellular response to oxidative stress^8,49^. To determine the involvement of NF-κB, we first investigated the localization of RelA/p65, the major NF-κB activating subunit, in our samples^50^. In serum-deprived DU145 and PC3 cells exposed to H_2_O_2_ (Fig. 5a,b), RelA/p65 was detected with higher nuclear expression via cellular fractionation compared to controls. Likewise, observations were mirrored via immunocytochemistry where RelA/p65 was primarily nuclear in serum-deprived DU145 and PC3 cells, and serum-deprived cell lines exposed to H_2_O_2_ (Fig. 5c,d). TNFα served as a positive control for nuclear localization of NF-κB^51,52^. Although a more complex mechanism is likely involved, this implicates the NF-κB-RELA/p65 pathway in contributing to the adaptability of cancer cells to oxidative stress environments.

### RelA/p65 (NF-κB) and quiescence cooperate during oxidative stress

**Figure 6:**
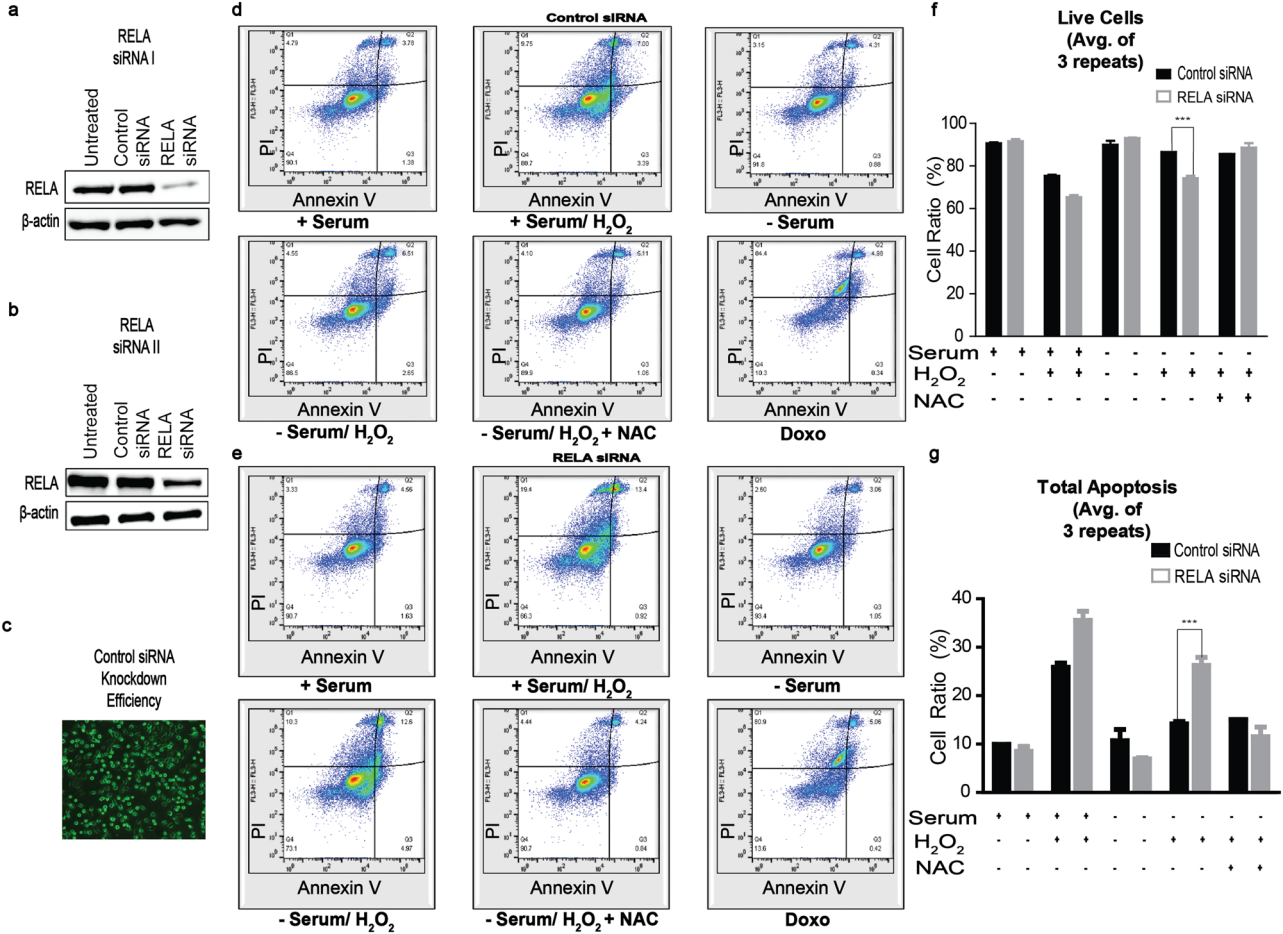
**RelA/p65 (NF-κB) knockdown promotes apoptosis in response to oxidative stress.** Two hundred thousand (2 × 10^5^) DU145 prostate cancer cells were plated in a 6-well dish and transfected with 100 nM human siRNA targeting RelA/p65 or nonspecific control siRNA for 24 h, followed by recovery in 10% FBS/RPMI for an additional 24 h. Cells were harvested for western blotting and apoptosis. (**a**, **b**) RelA/p65 was utilized to demonstrate that siRNA was specific as well as β-actin was used as a loading control to demonstrate equal amounts of protein was used for this assay. (**c**) Control siRNA efficiency was measured via fluorescence. Pictures of gels/blots were cropped to focus on target protein expression. Full length gels/blots are included in supplementary figure 5. (**d**, **e**) Cells were harvested with or without serum, then were treated with 250 μM H_2_O_2_, and/or 10 mM n-acetyl-cysteine (NAC) or 2 μM Doxorubicin (Doxo). One hundred thousand cells (1 × 10^5^) cells were measured via annexin V-FITC/PI double staining and flow cytometry. **Q1** (Annexin V+ , PI+): dead cells; **Q2** (Annexin V−, PI+): late stage apoptosis cells; **Q3** (Annexin V+ , PI −): early stage apoptosis cells; and **Q4** (Annexin V− , PI−): live cells. (**f**) A graphical representation of live cells. (**g**) A graphical representation of total apoptotic cells. Images were acquired via flow cytometry (Accuri C6 Cytometer; BD Biosciences); data was analyzed using FlowJo (v10). The mean ± SEM of data were obtained from three independent replicate experiments. Statistical analysis (*one-way ANOVA*) was done with GraphPad Prism (***p < 0.001; **p < 0.01; *p < 0.05).

**Figure 7:**
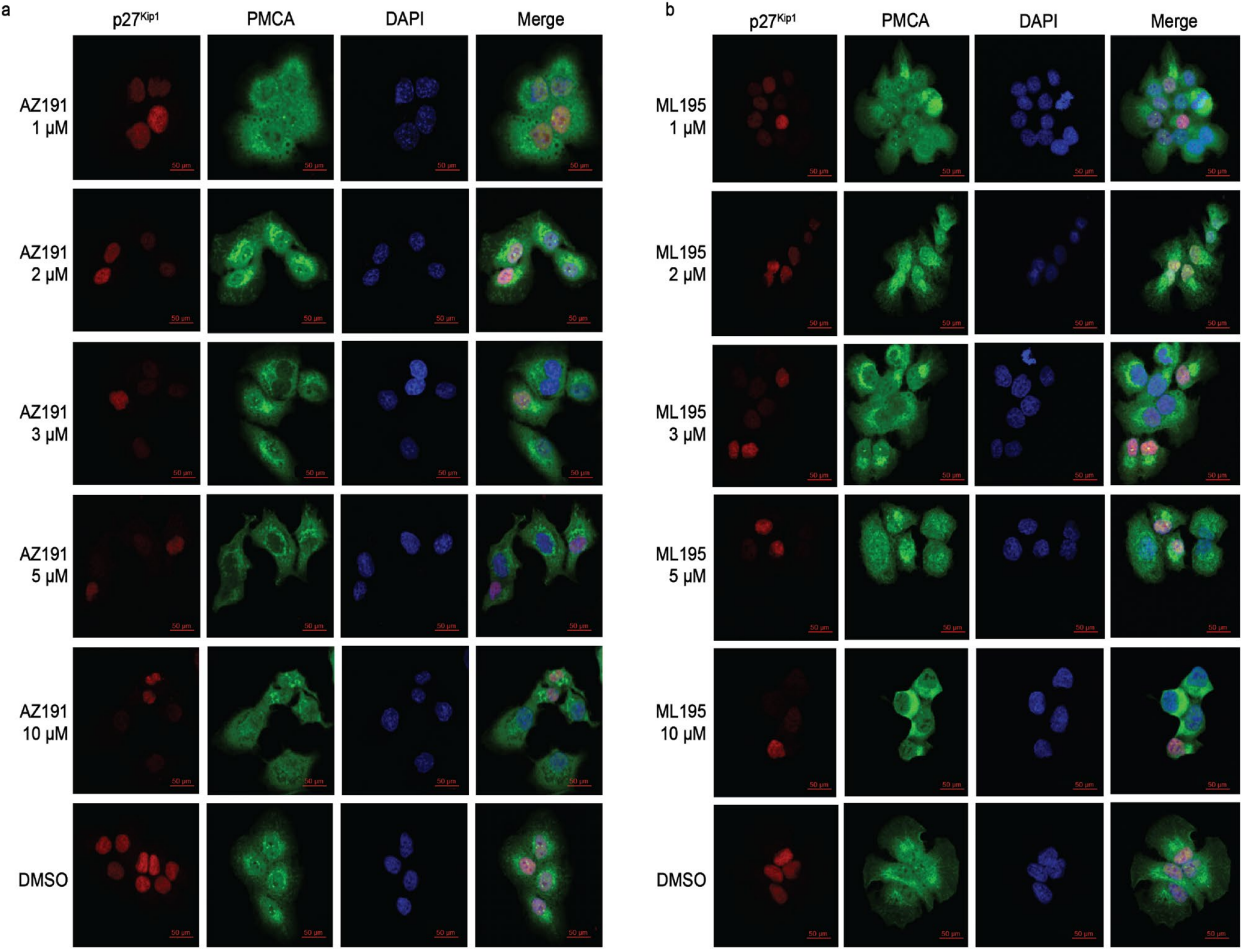
**Mirk/Dyrk1B inhibitors, AZ191 and NCGC00185981-05/ML195, inhibit oxidative stress-induced quiescence.** One hundred thousand (1 × 10^5^) DU145 cells were harvested for immunocytochemistry as described above prior to stimulating different concentrations (1, 2, 3, 5, and 10 µM) of Mirk/DYRK1B inhibitors: (**a**) AZ191 and (**b**) NCGC00185981-05/ML195 prior to culturing in serum-free media (24 h) stimulated with 250 μM H_2_O_2_ for 1 h to induce quiescence. Cells were then fixed with 4% paraformaldehyde, blocked, then incubated with an antibody mixture containing a rabbit p27^Kip1^ antibody and mouse anti-PMCA antibody, followed by secondary mixture containing a Cy3-conjugated anti-rabbit antibody and Alexa Fluor-488 anti-mouse antibody. DMSO served as a control. Imaging of cells (63×) was with a Zeiss LSM-700 Confocal Microscope. Scale bar = 50 μm.

**Figure 8:**
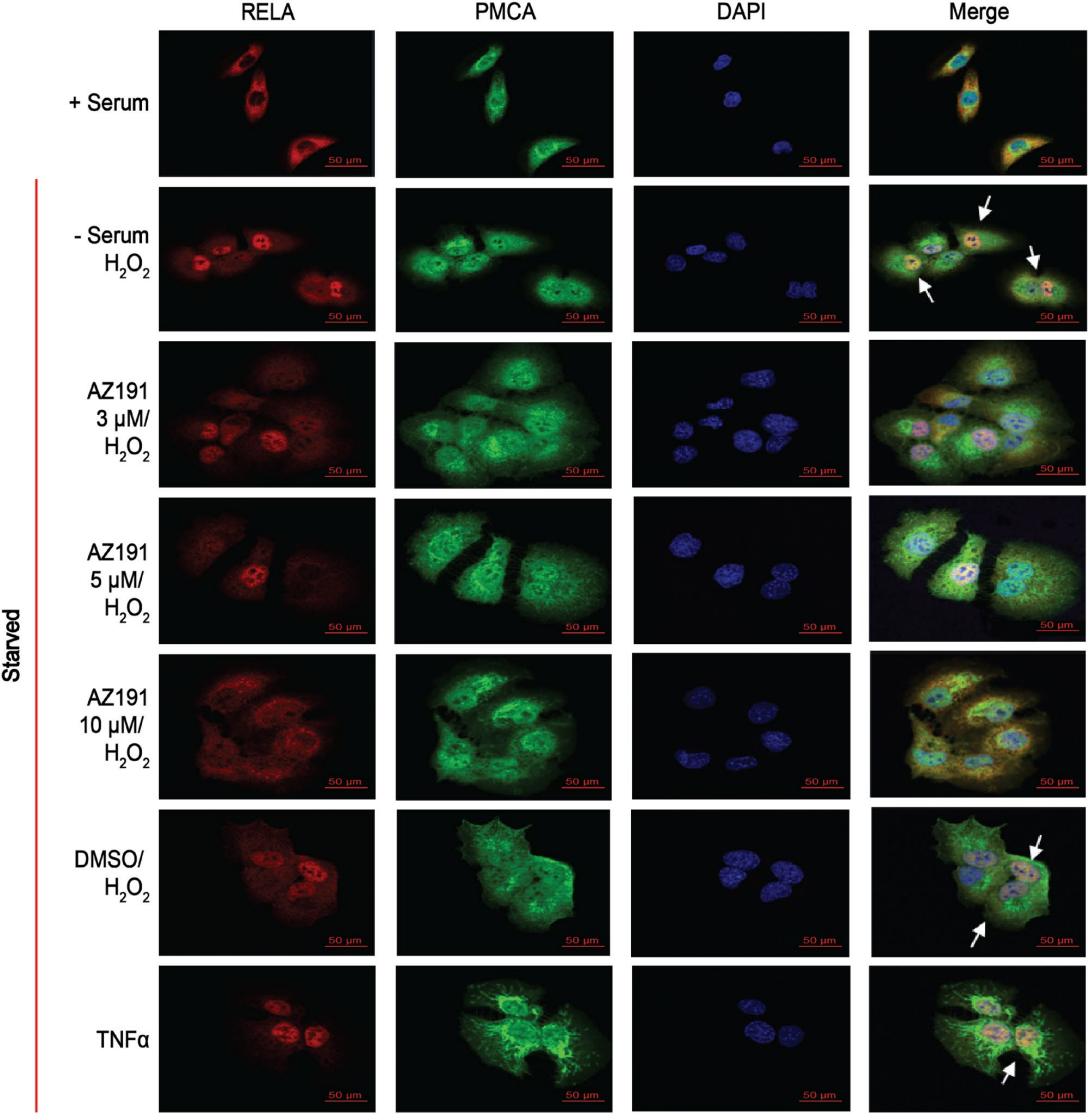
**Quiescence inhibitors prevented nuclear localization of RelA/p65 (NF-κB).** One hundred thousand (1 × 10^5^) DU145 prostate cancer cells were harvested for immunocytochemistry as described above plated on glass coverslips in 6-well plates. Cells were harvested with different concentrations (1, 2, 3, 5, and 10 µM) of AZ191. Cells were stimulated with different concentrations of AZ191 (3, 5, and 10 µM) of two different prior to culturing in serum-free media (24 h) stimulated with 250 μM H_2_O_2_ for 1 h to induce quiescence. Cells were then fixed with 4% paraformaldehyde, blocked, then incubated with an antibody mixture containing a rabbit NF-κB antibody and mouse anti-PMCA antibody, followed by secondary mixture containing a Cy3-conjugated anti-rabbit antibody and Alexa Fluor-488 anti-mouse antibody. DMSO was utilized as a control. Imaging of cells (63×) was with a Zeiss LSM-700 Confocal Microscope. Scale bar = 50 μm.

NF-κB is commonly associated with growth and the inflammatory response; however, some studies have indicated a role in quiescence^26,53,54^. Recently, literature has demonstrated NF-κB to be implicated in protecting cells from apoptosis^55–57^. To first assess whether NF-κB is required for oxidative stress adaptation, we transiently silenced RelA/p65 and examined the apoptosis profile upon exposure to ROS. Compared to prior results (Fig. 2b), the percentage of total dead DU145 cells significantly increased in serum-deprived cells exposed to H_2_O_2_ with the knockdown of RelA/p65 (Fig. 6). We did not observe this effect in PC3 cells where there was no change in apoptosis upon RelA/p65 knockdown. Initially, we observed a decrease in pRB protein expression and an accumulation of nuclear p27^Kip1^ in samples treated with TNFα, an inducer of RelA/p65 (NF-κB), suggesting a cooperation between NF-κB signaling and the quiescence program. Therefore, we sought to examine whether inhibition of quiescence reduced NF-κB-mediated cell survival during oxidative stress. We used two different quiescence inhibitors targeting Mirk/Dyrk1B (AZ191 and NCGC00185981-05/ML195) proteins regulating quiescence by stabilizing p27^Kip1^ phosphorylation and nuclear localization, and inducing the degradation of cyclin D^21,39^. We examined p27^Kip1^ nuclear accumulation in the presence of each inhibitor in DU145 prostate cancer cells (Fig. 7), and via immunocytochemistry, AZ191 (3–10 µM) was a more potent inhibitor of p27^Kip1^ nuclear expression (Fig. 7a) indicating that the cells were not quiescent. Likewise, we could not resolve a distinct nuclear localization of RelA/p65 with 5–10 µM AZ191 (Fig. 8), suggesting that a quiescent phenotype and NF-κB may synergistically protect tumor cells from oxidative stress.

## Discussion

Oxidative stress is considered to be one of the mechanisms that trigger early stages of prostate disease lesions, particularly prostatitic hyperplasia, benign prostatitic hyperplasia^58–60^, proliferative inflammatory atrophy (PIA)^61,62^, and others. Overall, if not subsided, the consequence(s) of oxidative stress result in a significant decrease in the antioxidant systems leading to lipid, protein, and DNA damage. However, at levels that are still under investigation, the resulting ROS during a stressful event can prime biochemical molecules to allow prostate cancer to develop and progress, such as deactivating tumor suppressors^35,63^ or increasing expression of pro-migratory signaling axes^35^. These dichotomic roles for ROS make it difficult to assess its clinical efficacy. For instance, questions that may arise during a clinical observation are: (1) is the observance of oxidative stress in a clinical BPH simply the result of aging tissue and vascular deterioration; (2) or is it the onset of tumor development; or (3) is the signaling potential of ROS collateral damage in cancer chemotherapy with the eventual outcome of a migratory tumor cell^34,64–67^. The preventative role of oxidative stress regulators is thought to protect the prostate from tumor development; however, chronic stress over time induces somatic mutations in DNA, lipids and proteins resulting in neoplastic transformation due to alterations in metabolic checkpoints. Additionally, the byproducts of ROS-based therapy are now being acknowledged to help propagate, amplify, and create a mutagenic and oncogenic microenvironment that is beneficial to a transforming metastatic tumor cell^68^. Thus, the metabolic relationships that are regulated by oxidative stress and the onset of prostate tumorigenesis remain an enigma.

In general, an advanced tumor is conditioned to survive in the poorest conditions i.e. when oxygen, glucose^69^ and amino acids^70^ are not accessible for metabolism during intravasation, extravasation and migration. Hypoxia and poor nutrition are common in a tumor mass due to insufficient vascularization of a heterogeneous and/or mobile tumor^71^. Angiogenesis is one of the hallmarks of survivability because neovascularization provides the nutrients and oxygen necessary for tumor sustainability. However, considering that tumors survive very well during the metastasis process and in a heterogeneous tumor mass with a limited vascular supply, there must be additional markers to access tumor survivability. Izuishi *et al.* posited two theoretical ways of adaptations to an insufficient oxygen and nutrient supply^72^. In brief, one way is by increasing the supply through angiogenesis, and the other way is by developing tolerance and alternate coping mechanisms to survive. Eventually, tumors grow beyond its ability to coordinate a vascular supply, and possibly, only the tumors cells that have learned to cope or adapt in a deprived environment might be the phenotype that advances to malignancy. Much therapeutic attention is paid to angiogenesis since this is a crucial event for survival and migration; however, therapeutic focus should expand to tumor populations that outgrow angiogenesis-dependent survival and can tolerate nutrient deprivation and oxidative stress.

We observed that starvation primed prostate cancer cells for further insult to oxidative stress. Although not investigated in this study, we have previously observed concomitant expression of HIF1-α and the chemotactic receptor, CXCR4, in DU145 prostate cancer cells under H_2_O_2_ oxidative stress^35,36^. Herein, our data support the notion that nutrient deprivation primes tumors for adaptation to stress and may serve as a determinant for tumor survival during stress and tumor progression^73^. Particularly, prostate cancer cells treated with both serum and H_2_O_2_ were apoptotic, a phenotype that was not observed in cells that were initially starved. Quiescent states allow diverse microorganisms to survive for long terms without nutrients in contrast to a proliferative “active” state that maintains itself by filtering out damaged molecules. During a state of quiescence, there must be a specialized protective biochemistry to counter damage for a cell to choose it as a first responding “safe house” to stress. For instance, Longo *et al.* reported that quiescent yeast cells retained a capacity to detect and respond to oxidative damage, making it a preferred phenotype during stress^72,74^. Additionally, Hill *et al.* demonstrated cellular stress induced a protective sleep-like state (quiescence) in *C. elegans, and* quiescence-defective organisms showed elevated expression of stress reporter genes and were impaired for survival, and suggest a deeply conserved function of quiescence^75^. Furthermore, quiescent and self-renewing stem cells easily reside in quiescence during hypoxia and oxidative stress^76,77^. Perhaps, this is what we are observing in cancer. Autophagy is another consideration for phenotypic adaptation to stress ^78^. Izuishi *et al.* demonstrated that some tumor cells acquired strong tolerance for nutrient deprivation. Pancreatic cancer cell lines, which are notoriously hypo-vascular as malignancy increases, survived for considerably longer periods under extremely low nutrient conditions than cell lines of liver cancer suggesting that tumor cells that have acquired the ability to survive an unfavorable microenvironment might be the most aggressive malignancy, and correlates with poor differentiation of tumors. In comparison, we observed that nutrient deprivation primed prostate cancer cells for tolerance of oxidative stress and adaptation for longer periods compared to cells with serum.

Adaptation and survival depended on a quiescent phenotype and transcription factor NF-κB which are usually responsive to low nutrient conditions and stress. Literature also suggests that NF-κB has alternate responses to oxidative stress depending on the context of the environment. While most studies focus on pro-apoptosis during times of stress^8^, less attention is paid to the anti-apoptotic functions of Rel/NF-κB complexes. In some situations, NF-κB promotes apoptosis in response to a specific cell type and stimulus^79–82^. For instance, Babaei *et al.* demonstrated that PC3 cells treated with biseugenol B, a cytotoxic agent, repressed the apoptosis-inhibitor activity of NF-κB thereby preventing NF-κB nuclear translocation^83^. Additionally, some Bcl-2 family member genes are up-regulated by NF-κB following the onset of stressful stimuli such as γ-irradiation and UV-radiation. Conversely, Bcl-2 has a reciprocal relationship with NF-κB whereby Bcl-2 has an inhibitory effect on NF-κB activity through stabilizing IkBa, inhibiting RelA transactivation, and interfering with the nuclear translocation of Rel family members^84,85^.

Early in our study, LNCaP demonstrated poor survivability during oxidative stress even when initially starved. We suspect that a p53 functional status determines the ability of ROS to induce different responses to death in LNCaP cells versus DU145 and PC3 cells. The LNCaP cell line has a wild-type, functional p53 while DU145 bears a mutant p53, and PC3 bears a frameshift producing a stop codon and an allele deletion^86^. The p53 transcription factor is a critical element in the cell’s ability to regulate the cell cycle and its response to DNA damage^86^. One of the most important unknowns in investigating p53 is how it determines a cellular outcome (cell cycle arrest vs. senescence vs. apoptosis) via regulation of outcome-specific target genes^87^. ROS act as both an up-stream signal that triggers p53 activation and as a downstream factor that mediates apoptosis^87^. Death is not the only outcome of p53 signaling during oxidative stress; however, Zhao *et al.* described that once in the mitochondria, p53 inhibits mitochondrial superoxide dismutase (MnSOD), playing a direct role in promoting apoptosis^88^. In addition, basal levels of p53 also has an antioxidant role, and the outcome depends on the context of the cell^88^.

How do cells recognize nutrient starvation? Izuishi *et al.* suggest that cells seem to recognize the amount of AMP and AMP-activated protein kinase in addition to stress signals for hypoxia, nutrient starvation, and physical stresses. Therefore, it is also probable that as cells increase in malignancy, they have already acquired constitutive tolerance for nutrient and oxygen starvation through multiple carcinogenesis steps^73^. Other considerations are a crosstalk between the mTORC1 and eIF2α pathways^89^, and AKT-mediated activation of NF-κB^90^. Early works attribute much of cellular survivability during oxidative stress to AKT^90^ where Song *et al.* described that inhibition of AKT phosphorylation induced decreases in sequential NF-κB signaling after 30 min of transient focal cerebral ischemia along with decreases in downstream survival signals of the AKT pathway. We also know that NF-κB activation and nuclear translocation can be blocked by PI3K/Akt inhibitors ^91^. In addition to the NF-κB/AKT relationship, MAPK is also involved in NF-κB signaling in a context dependent manner^92–94^. Moreover, in our own work, we demonstrated that ROS accumulation permitted AKT and CXCR4-mediated functions through PTEN catalytic inactivation. In this case, a disulfide bridge formed within the catalytic cleft of PTEN, inhibiting its suppressive functions, which allowed ROS to freely orchestrate signaling. We observed increased phosphorylated AKT (p-AKT) and CXCR4 expression, independent of any ligands, which were abrogated by a ROS scavenger in prostate cancer cells. ROS-mediated catalytic inactivation of PTEN did not affect its expression, yet enhanced cell migration and invasion in a CXCR4-dependent manner^35^. We also observed a contrary relationship between ROS and AKT where ROS facilitated cell death through activation of AKT. We initially observed that ROS increased expression of p-AKT in 22Rv1 human prostate cancer cells. The tumor suppressor, PTEN, a negative regulator of AKT signaling, was rendered catalytically inactive through oxidation by ROS, although the expression levels remained consistent. Despite these events, cells still underwent apoptosis. Further investigation into apoptosis revealed that expression of the tumor suppressor pVHL increased and contains a target site for p-AKT phosphorylation. pVHL and p-AKT associated in vitro, and knockdown of pVHL rescued HIF1α expression and the cells from apoptosis^36^. With all of this literature describing the relationship between ROS, AKT and cell survival, we believe our data is novel because there are no studies that describe the physical state of surviving epithelial tumor cells during an oxidative stress event. In literature, the phenom is reserved in cancer stem cells.

With regards to therapy, trans-arterial chemoembolization, damaging the blood supply of a tumor to prevent delivery of oxygen, growth factors, nutrients and others, has shown great success in providing significant improvement in overall survival, disease-free survival, and recurrence rates^95^. Although not considered curative, this intervention is dominant in liver cancer. The procedure is being considered for prostate cancer with hopes of causing irreversible necrosis of prostate tissue and causing the gland to shrink and soften^96,97^. This type of therapy would be the closest to nutrient deprivation therapy available for prostate lesions but would likely only be effective on a premalignant primary tumor mass versus a metastatic cells and/or cells that have adapted to nutrient deficiency; the challenge still remains in identifying tumors cells that are fully adapted to hostile conditions. Nevertheless, our results bring to the forefront a tumor cell phenotype that is often underappreciated, yet critical to metastasis, relapse and likely death.

## Methods

### Cell lines, antibodies, and reagents

Androgen-dependent LNCaP and androgen-independent DU145 and PC3 human tumor prostate cell lines with low, moderate, and high metastatic potential, respectively^98^, were purchased from American Type Culture Collection (ATCC) and maintained in complete either Lonza RPMI 1,640 (PC3 and LNCaP) media or Corning RPMI-1640 (DU145) supplemented with 10% FBS, 1% nonessential amino acids and 1% antibiotic–antimycotic. Cells were cultured at 37 °C and 5% CO_2_ and maintained at 80% confluency. Nuclear factor kappa-light-chain-enhancer of activated B cells (RelA/p65; WB: 1:1,000; ICC: 1:400), cyclin-dependent kinase inhibitor 1B (p27^Kip1^; WB: 1:1,000; ICC: 1:200), phosphorylated-retinoblastoma protein (pRB; 1:000) antibodies were purchased from Cell Signaling Technology. Plasma membrane-type Ca2^+^-ATPases (PMCA; ICC: 1:100), β-actin (1:1,000), and Topoisomerase I (1:1,000) antibodies were purchased from Santa Cruz Biotechnology. Doxorubicin (Doxo) was purchased from EMD Millipore and used at 2 µM working concentration. Human cytokine, tumor necrosis factor alpha (TNFα), was purchased from PeproTech, Inc. and used at 0.1 ng/mL working concentration. ROS scavenger, n-acetyl-cysteine (NAC), was purchased from Sigma-Aldrich and used at 5 mM and 10 mM working concentrations. Mirk/Dyrk1b inhibitor, AZ191 (used at 1, 2, 3, 5, 10 µM working concentrations), was purchased from Selleck Chem, and Mirk/Dyrk1b inhibitor, NCGC-00185981 (used at 1, 2, 3, 5, 10 µM working concentrations) was received as a gift from Edward Gellman, PhD. and Craig Thomas, PhD^99,100^.

### Chemical treatments

Prior to treatment with 250 μM hydrogen peroxide (H_2_O_2_), TNFα, NAC, and/or Mirk/Dyrk1b inhibitors, cells were serum-deprived for 24 h in serum-deprivation media (0.5% FBS, 0% non-essential amino acids, and 0% antibiotic/antimycotic) in 5% CO_2_ at 37 °C. Samples denoted as “untreated” or “control” received fresh media. Serum-deprived “control” samples were supplemented with DMSO vehicle for Mirk/Dyrk1b inhibitors.

### Phase-contrast microscopy

One hundred fifty thousand (1.5 × 10^5^) serum-containing and serum-deprived PC3, DU145, and LNCaP cells were seeded in 6-well plates. Serum-deprived cells were further stimulated with 250 μM H_2_O_2_ alone or pre-treated with 5 mM NAC for 1 h followed by stimulation with 250 μM H_2_O_2_ at different time points (0, 4, and 8 h). Light micrographs were taken at 10× and 20× magnification using a Zeiss Axiovert 200 M microscope.

### Measurement of ROS

Ten thousand (1 × 10^4^) serum-containing and serum-deprived PC3, DU145, and LNCaP cells were plated in black 96-well microplates. Cells were further stimulated with 250 μM H_2_O_2_ alone, 2 μM Doxorubicin, or pre-treated with NAC (5 mM) followed by stimulation with 250 μM H_2_O_2_. Treatment times varied per experiment (see figure legends). ROS generation was monitored using 2′,7′-Dichlorofluorescin diacetate (DCFDA; 10 μM^7,35,36^) via a microplate reader (Emission: 498 nm; Excitation: 522 nm, respectively). Experiments were performed at least thrice, and statistical analysis was performed with GraphPad Prism (***p < 0.001; **p < 0.01; *p < 0.05).

### Cell viability assay

A live/dead assay was conducted according to the manufacturer’s protocol (Abcam). Briefly, serum-containing and serum-deprived PC3, DU145, and LNCaP cells (1.5 × 10^5^) were harvested in a 6-well dish. Serum-deprived cells were further stimulated with 250 μM H_2_O_2_ for 4 h, or pre-treated with 5 mM NAC for 1 h prior to stimulation with 250 μM H_2_O_2_ for 4 h. Cells were then trypsinized, and cells (5 × 10^4^) were seeded in an 8-well chamber slide, and incubated in 1X Live and Dead dyes for 1 h at room temperature (RT) for 1 h. Cells were analyzed by fluorescent microscopy with a Zeiss Axio Vert. A1 Microscope. *Live:* emission at 495 nm; excitation at 488 nm. *Dead:* emission at 528 nm; excitation at 617 nm. Experiments were performed at least thrice, and statistical analysis was performed with GraphPad Prism (***p < 0.001; **p < 0.01; *p < 0.05).

### siRNA transfection

Transient transfection of 100 µM RelA/p65-specific human siRNA (Cell Signaling Technology) was performed on DU145 cells using Lipofectamine 2000 (Invitrogen). One hundred fifty thousand (1.5 × 10^5^) cells were seeded in 10% FBS/RPMI in 6-well culture plates and then transfected with RelA/p65 or scramble/control-siRNA (Cell Signaling Technology and Santa Cruz Technology) in Opti-MEM at 37 °C and 5%CO_2_ for 24 h. Cells were recovered in 10% FBS/RPMI for an additional 24 h, followed by serum-deprivation for 24 h. Serum-containing and serum-deprived cells were further stimulated (alone or in combination) with 250 μM H_2_O_2_, 10 mM NAC or 2 μM Doxorubicin for 24 h.

### Annexin-V apoptosis assay

Annexin-V Apoptosis Detection Kit (Cell Signaling Technology) was used to quantify the levels of apoptosis according to the manufacturer's instructions. Briefly, one million (1.0 × 10^6^) serum-containing and serum-deprived PC3, DU145, and LNCaP cells were stimulated (alone or in combination) with 250 μM H_2_O_2_, 10 mM NAC or 2 μM Doxorubicin for 24 h. Cells were trypsinized and harvested for 15 min incubation with Annexin V-FITC and propidium iodide. Apoptosis was analyzed by flow cytometry (Accuri C6, BD Biosciences) for the detection of Annexin V-FITC. Data was analyzed using FlowJo (v10). Experiments were performed at least thrice, and statistical analysis was performed with GraphPad Prism (***p < 0.001; **p < 0.01; *p < 0.05).

### Cell proliferation assay

To measure cell proliferation, an EdU (5-ethynyl-2′-deoxyuridine) Proliferation Kit (Abcam) was employed according to the manufacturer’s protocol. One million (1.0 × 10^6^) serum-containing and serum-deprived PC3 and DU145 were stimulated as described above prior to the addition of 20 μM EdU for 3 h. Alternatively, control cells were incubated in 10% FBS without EdU label at 37 °C for 24 h. Cells were fixed with 3.7% formaldehyde in 1 × PBS at RT for 15 min followed by permeabilization with 0.5% Triton X-100 in 1 × PBS for 20 min at RT. Cells were incubated in reaction cocktail for 30 min at RT then washed twice with 1 × PBS. Cells were analyzed by flow cytometry (Accuri C6, BD Biosciences) for the detection of EdU-positive (EdU^+^) cells. Data was analyzed using FlowJo (v10). Experiments were performed at least thrice, and statistical analysis was performed with GraphPad Prism (***p < 0.001; **p < 0.01; *p < 0.05).

### Subcellular fractionation

Subcellular fractionation technique was performed as we’ve previously described^101^. Two million (2.0 × 10^6^) serum-containing and serum-deprived PC3 and DU145 cells were stimulated with 250 μM H_2_O_2_ or TNFα (0.1 ng/mL) for 30 min. Alternatively, select samples were pre-treated with 5 mM NAC for 1 h prior to 250 μM H_2_O_2_ for 1 h. Cells were harvested for subcellular fractionation according to the manufacture’s protocol (NE-PER Nuclear and Cytoplasmic Extraction Kit, ThermoFisher). Briefly, cells were lysed in a series of buffers, centrifuged to obtain a non-nuclear fraction and an intact nuclear pellet, and further lysed to isolate the nuclear fraction. Forty micrograms (40 μg) of total cell lysate were resolved by SDS-PAGE to detect RelA/p65 (1:1,000, Cell Signaling Technology). Topoisomerase I (1:1,000, Santa Cruz Biotechnology) and β-actin (1:1,000, Cell Signaling Technology) were used as loading controls.

### Immunocytochemistry

Immunocytochemistry technique was performed as we’ve previously described^101^. One hundred thousand (1 × 10^5^) serum-containing and serum-deprived PC3 and DU145 cells were plated on glass coverslips (Fisher). Cells were stimulated as previously described above. Cells were fixed with 4% paraformaldehyde for 40 min at RT and washed with 1 × PBS and 0.1% Tris–glycine. Non-specific proteins were blocked in blocking solution (5% normal donkey serum/1% BSA/0.3% Triton X-100 in 1 × PBS) for 30 min at RT, prior to incubating with RelA/p65 (1:400, Cell Signaling Technology), p27^Kip1^ (1:200, Cell Signaling Technology), or PMCA (1:100, Santa Cruz Biotechnology) in blocking solution at 4 °C overnight. Secondary detection was with Cy3-conjugated donkey anti-rabbit IgG (1:200–400, Jackson ImmunoResearch), or FITC-conjugated anti-mouse IgG (1:100, Jackson ImmunoResearch), or Alexa Fluor 488 dye (1∶500, Invitrogen) in blocking solution at RT for 1 h followed by three washes in 1 × PBS. Nuclei were detected with DAPI in 1 × PBS prior to mounting in Aqua-Polymount (Polyscience, Inc). Images were taken with Zeiss LSM-700 Confocal Microscope at excitation 488 nm for Alexa Fluor and 550 nm for Cy3.

### Immunoblotting

Immunoblotting technique was performed as we’ve previously described^102^. Briefly, one million (1.0 × 10^6^) serum-containing or serum-deprived cells were stimulated with 250 μM H_2_O_2_ for 1 h or TNFα (0.1 ng/mL) for 30 min. Alternatively, cells were pre-treated with 5 mM NAC for 1 h followed by 250 μM H_2_O_2_ for 1 h. Cells were lysed and sonicated in 1 × Cell Signaling Technology lysis buffer prior to incubation on ice for 30 min. Lysates were centrifuged at max speed for 10 min at 4 °C, and then equal amounts of protein per sample were separated by SDS-PAGE and transferred to PVDF membrane. Protein bound membranes were blocked in 5% BSA/1XTBST and subsequently incubated with primary antibodies p27^Kip1^ (1:1,000, Cell Signaling Technology) or pRB (1:1,000, Cell Signaling Technology) overnight at 4 °C in 5% BSA/TBST. Beta actin (β-actin; 1:1,000; Santa Cruz Biotechnology) served as a loading control. Primary antibodies were detected by HRP-conjugated secondary antibodies (1:10,000, Jackson ImmunoResearch) diluted in 5% BSA/1 × TBST. Protein expression was detected with chemiluminescence (Luminata Western HRP Chemiluminescence Substrates; Millipore Sigma) on ChemiDoc MP Imaging System (Bio-Rad, USA).

### Statistical analysis

Statistical analyses and graphs were generated using GraphPad Prism 6.0 software (San Diego, CA, USA) ***p < 0.001; **p < 0.01; *p < 0.05 were considered significant.

## Supplementary information

Supplementary information

## Data Availability

The data that support the findings of this study are available from the corresponding author upon reasonable request.
