# Supplementary material for: Serum deprivation initiates adaptation and survival to oxidative stress in prostate cancer cells
Source: Sci Rep. 2020 Jul 27;10:12505. doi: 10.1038/s41598-020-68668-x (PMC7385110; doi:10.1038/s41598-020-68668-x)
Supplement: Supplementary file 1 — Supplementary information [file 41598_2020_68668_MOESM1_ESM.pdf]

Serum deprivation initiates adaptation and survival to oxidative stress in prostate cancer cells

ElShaddai Z. White[1,2], Nakea M. Pennant[1,2], Jada R. Carter[1,2], Ohuod Hawsawi[3], Valerie Odero-Marah[1,2], Cimona V. Hinton[1,2]*
[1]Department of Biological Sciences, Clark Atlanta University (CAU), Atlanta, GA 30314, USA
[2]Center for Cancer Research and Therapeutic Development, Clark Atlanta University (CAU), Atlanta, GA 30314, USA
[3]Department of Molecular and Human Genetics, Baylor College of Medicine, Houston, TX 77030, USA
*Correspondence: chinton@cau.edu

PC3

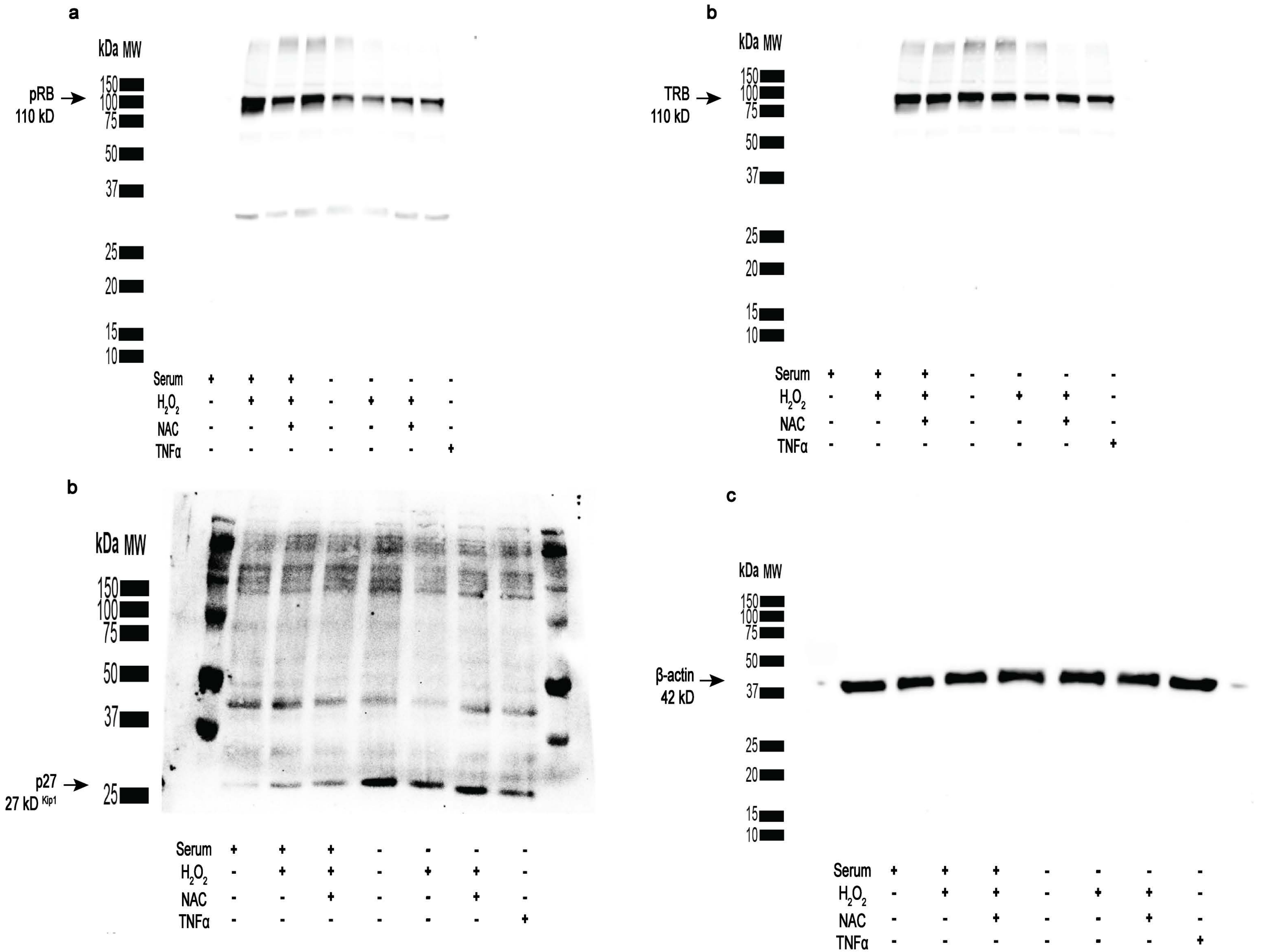
a
kDa MW
pRB
110 kD
150
100
75
50
37
25
20
15
10
Serum + + + - - - -
H2O2 - + + - + + -
NAC - - + - - + -
TNFα - - - - - - +
b
kDa MW
TRB
110 kD
150
100
75
50
37
25
20
15
10
Serum + + + - - - -
H2O2 - + + - + + -
NAC - - + - - + -
TNFα - - - - - - +
b
kDa MW
150
100
75
50
37
25
p27
27 kD Kip1
Serum + + + - - - -
H2O2 - + + - + + -
NAC - - + - - + -
TNFα - - - - - - +
c
kDa MW
150
100
75
50
37
25
20
15
10
β-actin
42 kD
Serum + + + - - - -
H2O2 - + + - + + -
NAC - - + - - + -
TNFα - - - - - - +

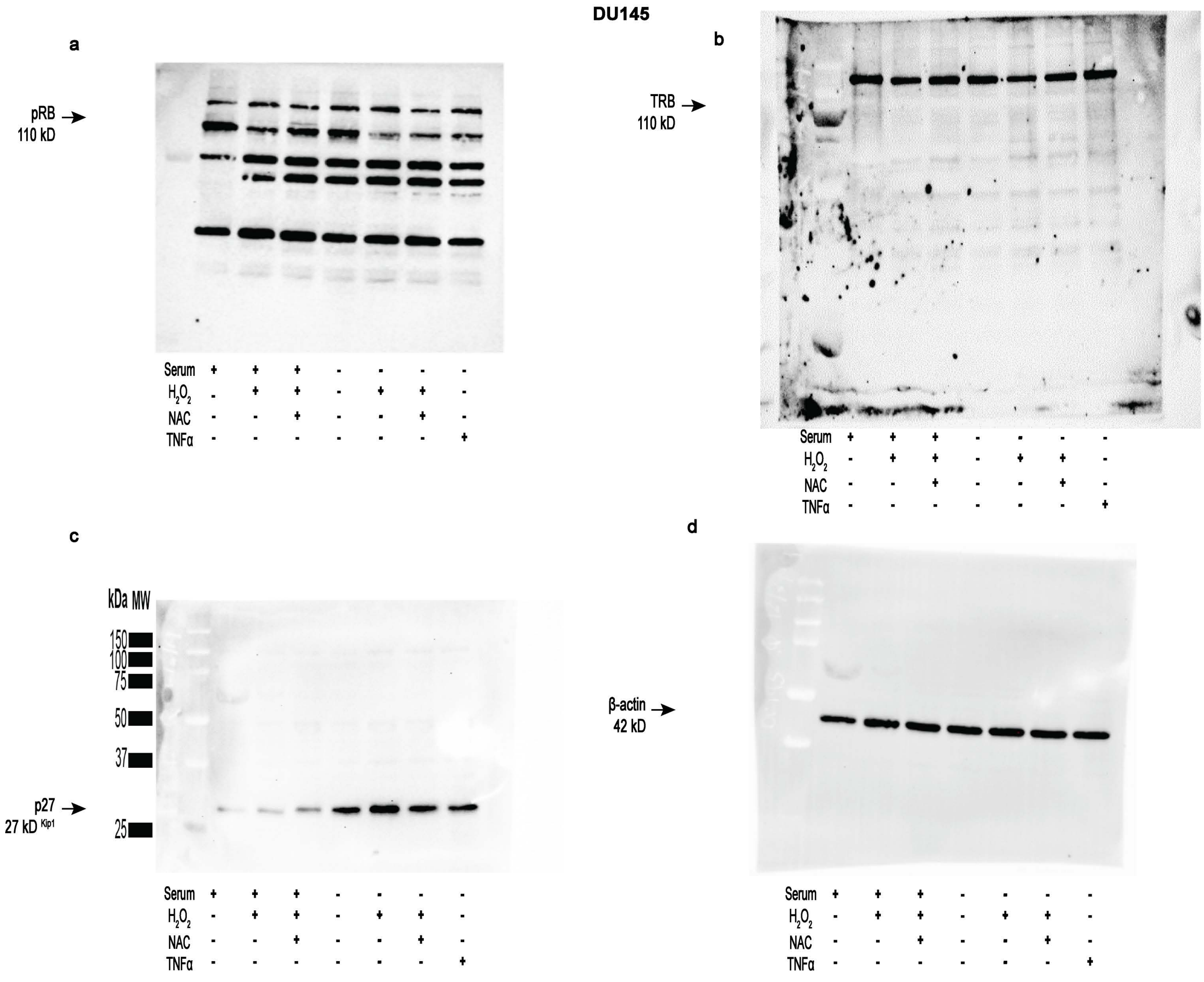
DU145
a
pRB
110 kD
Serum + + + - - - -
H2O2 - + + - + + -
NAC - - + - - + -
TNFα - - - - - - +
b
TRB
110 kD
Serum + + + - - - -
H2O2 - + + - + + -
NAC - - + - - + -
TNFα - - - - - - +
c
kDa MW
150
100
75
50
37
25
p27
27 kD Kip1
Serum + + + - - - -
H2O2 - + + - + + -
NAC - - + - - + -
TNFα - - - - - - +
d
β-actin
42 kD
Serum + + + - - - -
H2O2 - + + - + + -
NAC - - + - - + -
TNFα - - - - - - +

PC3

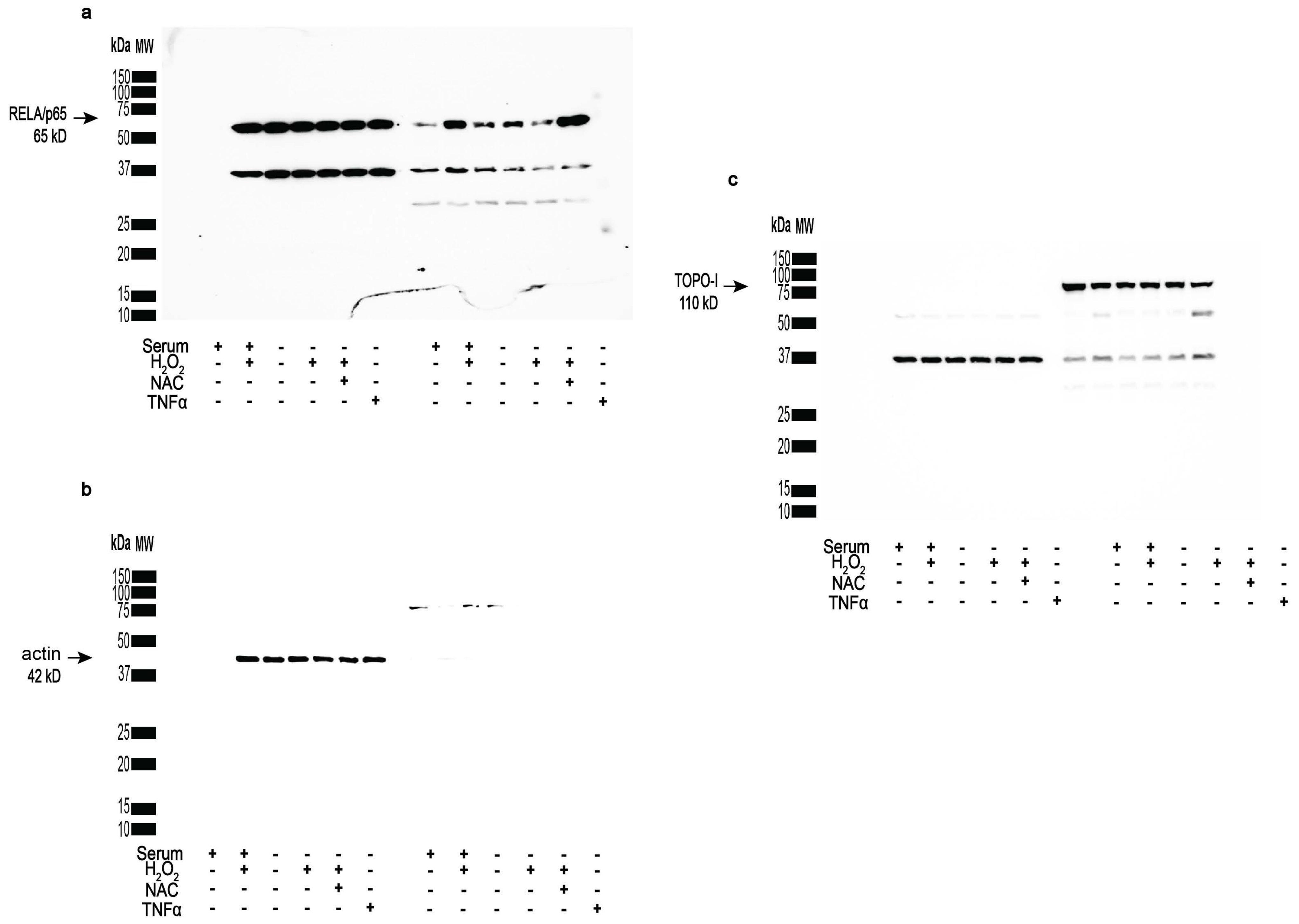
a
kDa MW
150
100
75
50
37
25
20
15
10
RELA/p65
65 kD
Serum + + - - - - + + - - - -
H_2O_2 - + - + + - - + - + + -
NAC - - - - + - - - - - + -
TNFα - - - - - + - - - - - +
b
kDa MW
150
100
75
50
37
25
20
15
10
actin
42 kD
Serum + + - - - - + + - - - -
H_2O_2 - + - + + - - + - + + -
NAC - - - - + - - - - - + -
TNFα - - - - - + - - - - - +
c
kDa MW
150
100
75
50
37
25
20
15
10
TOPO-I
110 kD
Serum + + - - - - + + - - - -
H_2O_2 - + - + + - - + - + + -
NAC - - - - + - - - - - + -
TNFα - - - - - + - - - - - +

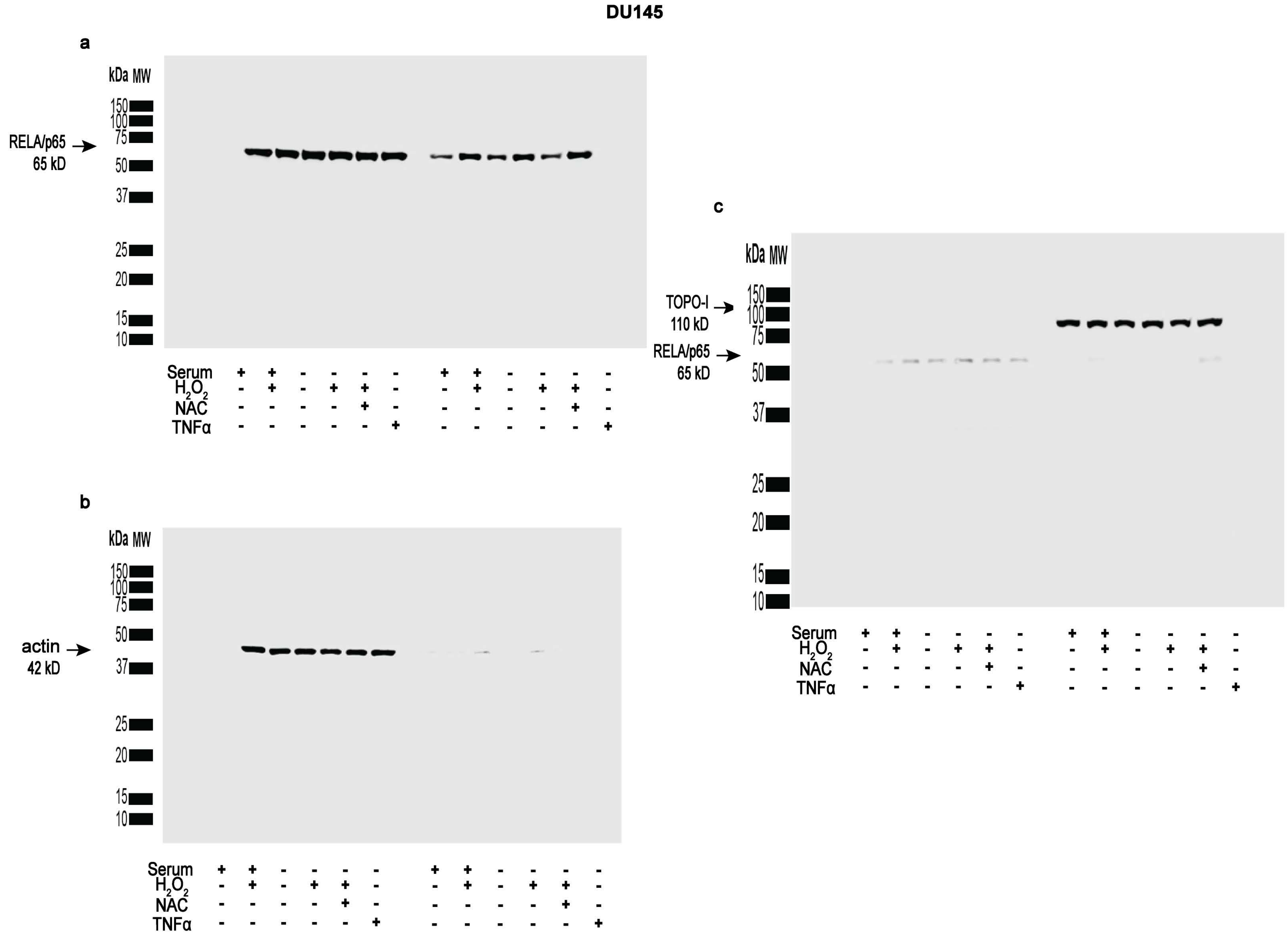
DU145
a
kDa MW
150
100
75
50
37
25
20
15
10
RELA/p65
65 kD
Serum
H2O2
NAC
TNFα
b
kDa MW
actin
42 kD
c
kDa MW
TOPO-I
110 kD
RELA/p65
65 kD

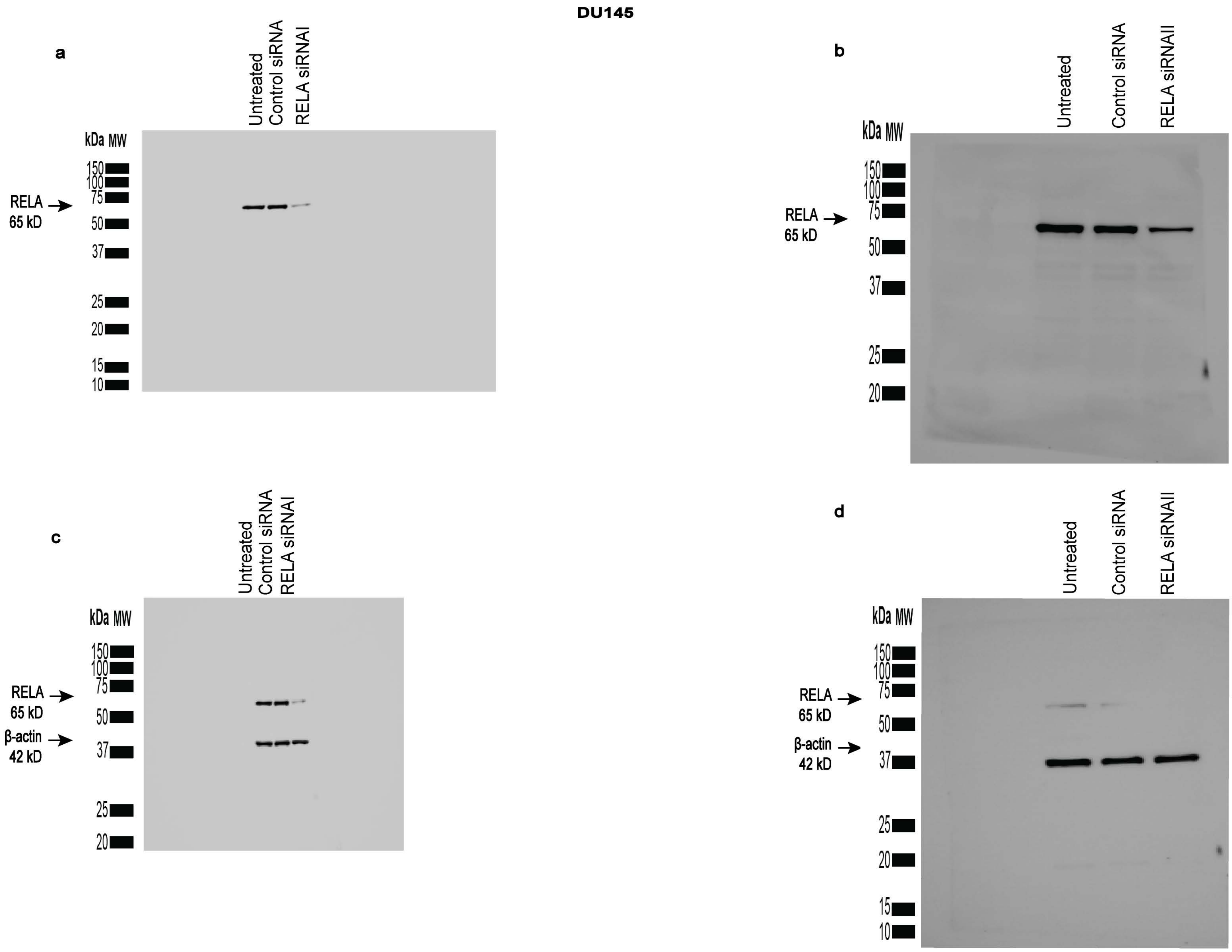
DU145
a
Untreated
Control siRNA
RELA siRNAI
kDa MW
150
100
75
50
37
25
20
15
10
RELA 65 kD
b
Untreated
Control siRNA
RELA siRNAII
kDa MW
150
100
75
50
37
25
20
RELA 65 kD
c
Untreated
Control siRNA
RELA siRNAI
kDa MW
150
100
75
50
37
25
20
RELA 65 kD
β-actin 42 kD
d
Untreated
Control siRNA
RELA siRNAII
kDa MW
150
100
75
50
37
25
20
15
10
RELA 65 kD
β-actin 42 kD

Supplemental Figure 1. Full length gel/blot demonstrating molecular-weight for immunoblots in Figure 4a. (a) Full-length gel/blot depicting protein expression of phosphorylated-retinoblastoma protein (pRB) in PC3 prostate cancer cells. (b) Full-length gel/blot depicting protein expression of total-retinoblastoma protein (TRB) in PC3 prostate cancer cells. (c) Full-length gel/blot depicting protein expression of $p27_{Kip1}$ in PC3 prostate cancer cells. (d) Full-length gel/blot depicting protein expression of loading control, β-actin.

Supplemental Figure 2. Full length gel/blot demonstrating molecular-weight for immunoblots in Figure 4b. (a) Full-length gel/blot depicting protein expression of phosphorylated-RB (pRB) in DU145 prostate cancer cells. (b) Full-length gel/blot depicting protein expression of total-retinoblastoma protein (TRB) in DU145 prostate cancer cells. (c) Full-length gel/blot depicting protein expression of $p27_{Kip1}$ in DU145 prostate cancer cells. (c) Full-length gel/blot depicting protein expression of loading control, β-actin.

Supplemental Figure 3. Full length gel/blot demonstrating molecular weight for immunoblots in Figure 5. (a) Full-length gel/blot depicting cytoplasmic and nuclear fractions protein expression of RELA/p65 (NF-κB) in PC3 prostate cancer cells. (b) Full-length gel/blot depicting protein expression of β-actin cytoplasmic loading control. (c) Full-length gel/blot depicting protein expression of Topoisomerase I (TOPO-I) nuclear loading control.

Supplemental Figure 4. Full length gel/blot demonstrating molecular weight for immunoblots in Figure 5. (a) Full-length gel/blot depicting cytoplasmic and nuclear fractions protein expression of RELA/p65 (NF-κB) in DU145 prostate cancer cells. (b) Full-length gel/blot depicting protein expression of β-actin cytoplasmic loading control. (c) Full-length gel/blot depicting protein expression of Topoisomerase I (TOPO-I) nuclear loading control.

Supplemental Figure 5. Full length gel/blot demonstrating molecular weight for immunoblots in Figure 6. (a) Full-length gel/blot depicting protein expression of RelA/p65 (NF-κB) in RelA/p65 (NF-κB) siRNA I. (b) Full-length gel/blot depicting protein expression of of loading control, β-actin. (c) Full-length gel/blot depicting protein expression of RelA/p65 (NF-κB) in RelA/p65 (NF-κB) siRNA II. (d) Full-length gel/blot depicting protein expression of loading control, β-actin.
